# Fruit Seeds as Sources of Bioactive Compounds: Sustainable Production of High Value-Added Ingredients from By-Products within Circular Economy

**DOI:** 10.3390/molecules24213854

**Published:** 2019-10-25

**Authors:** Marina Fidelis, Cristiane de Moura, Tufy Kabbas Junior, Nora Pap, Pirjo Mattila, Sari Mäkinen, Predrag Putnik, Danijela Bursać Kovačević, Ye Tian, Baoru Yang, Daniel Granato

**Affiliations:** 1 MSc in Food Science and Technology, Ponta Grossa 84035010, Brazil; marinafidelis01@gmail.com; 2 Graduate Program in Chemistry, State University of Ponta Grossa, Avenida Carlos Cavalcanti, 4748, Ponta Grossa 84030900, Brazil; tiane.moura@hotmail.com (C.d.M.); tufy_kj@hotmail.com (T.K.J.); 3 Food Processing and Quality, Innovative Food System, Production Systems Unit, Natural Resources Institute Finland (Luke), Tietotie 2, FI-02150 Espoo, Finland; nora.pap@luke.fi (N.P.); pirjo.mattila@luke.fi (P.M.); sari.makinen@luke.fi (S.M.); 4 Faculty of Food Technology and Biotechnology, University of Zagreb, Pierottijeva 6, 10000 Zagreb, Croatia; pputnik@alumni.uconn.edu (P.P.); dbursac@pbf.hr (D.B.K.); 5 Food Chemistry and Food Development Unit, Department of Biochemistry, University of Turku, FI-20014 Turku, Finland; yetiang@gmail.com (Y.T.); bayang@utu.fi (B.Y.)

**Keywords:** functional ingredients, food waste, waste management, *Myrciaria*, *Vitis*, antiproliferative agents, horizontal economy, antioxidant activity, phenolic compounds, oil

## Abstract

The circular economy is an umbrella concept that applies different mechanisms aiming to minimize waste generation, thus decoupling economic growth from natural resources. Each year, an estimated one-third of all food produced is wasted; this is equivalent to 1.3 billion tons of food, which is worth around US$1 trillion or even $2.6 trillion when social and economic costs are included. In the fruit and vegetable sector, 45% of the total produced amount is lost in the production (post-harvest, processing, and distribution) and consumption chains. Therefore, it is necessary to find new technological and environmentally friendly solutions to utilize fruit wastes as new raw materials to develop and scale up the production of high value-added products and ingredients. Considering that the production and consumption of fruits has increased in the last years and following the need to find the sustainable use of different fruit side streams, this work aimed to describe the chemical composition and bioactivity of different fruit seeds consumed worldwide. A comprehensive focus is given on the extraction techniques of water-soluble and lipophilic compounds and *in vitro/in vivo* functionalities, and the link between chemical composition and observed activity is holistically explained.

## 1. Introduction

According to Homrich et al. [1], the circular economy (CE) is an umbrella concept that applies different mechanisms aiming to minimize waste generation, thus decoupling economic growth from natural resources. European Union (EU) countries are leading this concept by promoting the responsive and cyclical use of resources and contributing to sustainability [2]. From the economic standpoint, CE is a model that should replace the conventional, linear material, and energy flow models by addressing the issues of environmental deterioration, social equity, and long, sustainable economic growth [3]. In fact, a holistic assessment based on a dashboard of qualitative and quantitative indicators is necessary to encompass the environmental, economic, social, and technical dimensions of CE [2,4].

Much discussion has been dedicated to the true definitions and scopes of CE, yet it is not fully comprehended in quantitative terms according to Moraga et al. [2]. According to the United Nations Sustainable Development Goals, CE can improve social equity, promote economic growth, and permanently reduce the rate of extraction of raw materials by closing material loops [5,6]. According to these goals, more specifically *Goal 9* and *Goal 12*, the technological progress is the foundation of efforts to achieve environmental objectives, such as increased resource and energy efficiency. There needs to be more investment in high-tech manufacturing for the increased efficiency and decreased waste for a sustainable and responsible consumption of natural resources. Indeed, net welfare gains from economic activities can increase by reducing resource use, degradation, and pollution along the entire life cycle in a food supply chain, while increasing the quality of life [7].

According to Slorach et al. [8], each year, an estimated one-third of all food produced is wasted; this is equivalent to 1.3 billion tons of food, which is worth around US$1 trillion or even $2.6 trillion when social and economic costs are included. This amount of food ends up rotting in the bins of consumers and retailers or spoiling due to poor transportation and harvesting practices. Following the CE principles and directives, ideally, this should be fully avoided, or if not possible, recycled to recover resources [8]. Food and pharmaceutical companies should decisively work on identifying niches within the value chains where such interventions have the greatest potential to improve the environment and enhance the social impacts of production, while integrating the economic feasibility in the production and consumption chains [9].

From 2007 to 2017, the largest fruit producers were China, India, Brazil, the USA, Turkey, Mexico, Spain, Indonesia, Italy, and the Philippines [10], in total accounting for more than 810 million metric tons of produce. The most produced items were: watermelons (118 million tons), bananas (114 million tons), apples (83 million tons), grapes (74 million tons), oranges (73 million tons), mango (51 million tons), tangerines (33 million tons), pineapple (27 million tons), peaches and nectarines (25 million tons), pears (24 million tons), lemons and limes (17 million tons), papaya (13 million tons), and plums/sloes (12 million tons) [11]. In the fruit and vegetable sector, 45% of the total production is lost in post-harvest, processing, distribution, and consumptions chains [12]. Therefore, it is necessary to find new technological and environmentally friendly solutions to utilize fruit wastes as new raw materials and develop scale-ups for the production of high value-added products [13,14,15,16,17].

Searching for solutions based on CE requirements, Sagar et al. and Ayoub et al. [18,19] reviewed the extraction technologies, bioactive compounds, and possible utilization of fruit and vegetable waste. According to these authors, on average, 25–30% of wastes are generated in the processing of fruits and vegetables, but this number can even be in a higher range of 30–50% for mangos and oranges, and up to 70% for durian, jackfruit, and mangosteen.

So far, the literature has proposed the use of fruit wastes in the development of new products as functional extracts and flours from grape waste [20,21], yogurts enriched with grape skin flour [22], cookies added with grape skin and grape seed flours [23], fish oil added with apple peel phenolics [24], avocado oil added with phenolic compounds [25], frozen yogurt containing sea buckthorn berry pomace [26], bologna sausage added with citrus fiber [27], meat products added with berry extracts [28], corn starch extrudate containing fruit pomace (cranberry, grape, apple, and blueberry) [29], oil-in-water emulsion containing Ander berry [30], and Dijon-style mustard added with berry fruit pomace [31], among other technological applications. Surprisingly enough, the peels and seeds of the most conventionally grown fruits and vegetables are not yet used to develop new foods, natural extracts, and pharmaceutical products [32,33,34,35]. This is probably because of the remarkable presence of pesticide residues in the peels [36].

Not only technological applications containing fruit side streams have been studied, but also the optimization of extraction parameters aiming to increase the level of bioactive compounds, namely phenolic compounds and carotenoids, to produce bioactive-rich extracts and then further add into different foods [37]. For instance, Takii et al. [38] studied the effects of ethanol (EtOH), extraction time, and temperature on the extraction rate of phenolic compounds and antioxidants from banana peel, whereas Wong et al. [39] optimized the extraction conditions of total phenolic content and antioxidant activity of passion fruit peel using the same factors (e.g., EtOH concentration in the extracting solution, time, and temperature). In the same line of research, Dorta et al. [40] assessed the effects of EtOH, water, and acetone as solvents and different temperatures on the extraction of antioxidants from mango peel and seeds. Table 1 contains some studies [15,28,41,42,43,44,45,46,47,48,49,50,51,52,53,54,55,56,57,58,59,60,61,62,63,64,65,66,67,68,69] regarding the extraction of bioactive compounds from fruit seeds and the bioactivity already studied. It is clear that new studies regarding fruit seeds should be focused on the optimization of extraction conditions and on the extraction techniques so that new value-added extracts can be potentially used as ingredients by pharmaceutical and food sectors. The extraction methods are highlighted to provide useful information about new techniques that can be explored industrially for scale-up process, and therefore enlarge the use of natural side streams as raw materials to obtain bioactive-rich extracts. Aside from that, conventional extracting techniques, such as maceration, are time consuming, which can limit its applicability in the industrial sector.

Considering that the production and consumption of fruits has increased over the last years and following the need to find sustainable uses of underutilized fruit byproducts, this work aimed to describe the chemical composition and bioactivity of different fruit seeds consumed worldwide. A comprehensive focus is given on the extraction techniques of water-soluble and lipophilic compounds and *in vitro/in vivo* functionalities, and the link between chemical composition and observed activity is holistically explained.

## 2. Fruit By-Products (Seeds): Chemical Characterization and Bioactivities

### 2.1. Apple (Pyrus malus *L.*)

Apples are mainly consumed fresh; however, 25–33% of fruits are processed into juice, cider, jams, and other products. Globally, the most used processed apple product is the juice. The recovery of juice in the industrial processing is around 70–75%, whereas 25–30% *w/w* of apple pomace is generated. Seeds account for 2–4% *w/w* of the pomace [11,74]. Considering the massive production of apples, seeds represent a significant side stream.

The oil content of apple seeds varies from 17% to 29% *v/w*, and the main fatty acids are oleic (27.0–46.5% *w/w*) and alpha linoleic acid (43.8–60.0% *w/w*). Other prominent fatty acids are palmitic, stearic, and arachidic acids [75,76,77,78,79]. A high percentage of unsaturated fatty acids makes apple seed oil nutritionally favorable, having positive effects on lowering low-density lipoproteins (LDL) cholesterol and preventing risks of cardiovascular diseases [79]. Apple seed oil is also a rich source of tocopherols, sterols, and phospholipids [76,77,80]. According to Tian et al. [78], apple seed oil is active against bacteria, mildews were less sensitive to apple seed oil than yeasts, and the minimum inhibitory concentration (MIC) of apple seed oil ranged from 0.3 to 0.6 mg/mL. The observed biological activities showed that the oil had a good potential for uses in the food industry and pharmacy.

The nutritional value of protein from defatted apple seed meal was relatively low in the murine study performed by Opyd et al. [81]. However, the protein content of apple seeds is quite high, varying from 34 to 50% *w/w*, and it is rich in sulfur-containing amino acids. The fiber content of seeds is 3.9–4.3% *w/w*. Seeds also contain significant amounts of minerals such as phosphorus, potassium, magnesium, calcium, and iron, in the order of 720, 650, 510, 210 and 110 mg/100 g, respectively [78,79]. 

Apple seeds are a good source of polyphenols. According to Xu et al., Lu and Yeap Foo et al., and Schieber et al. [50,71,72], the seeds of apples contain phenolic acids (protocatechuic, chlorogenic, coumaric, ferulic, and caffeic acids), quercetin derivatives, (+)-catechin, (-)-epicatechin, proanthocyanin B2, and phloridzin. Phloridzin is the predominant phenolic compound, and its content has been reported to be 192 mg/100 g dry weight (DW) in dried apple seeds [71], 240–864 mg/100 g DW in the seeds of seven apple cultivars [50], 326–2235 mg/100 g in dried defatted apple seeds of 12 dessert and cider cultivars [52], and 64 mg/100 g DW in the seeds of Ida red cultivar [57]. Phloridzin contents are higher in seeds than in peels and pomaces [50,57].

Apple phytochemicals and especially phloridzin may have many beneficial health effects. It has anti-inflammatory and antioxidant activities, and it reduces hyperglycemia by blocking renal glucose resorption and intestinal glucose absorption through inhibition of the sodium–glucose symporters [38,82,83,84,85]. According to the more recent murine study by Shin et al. [86], phloridzin may prevent diet-induced obesity, hepatic steatosis, inflammation, fibrosis, and insulin resistance. Phloridzin can be potentially used as treatment for type 2 diabetes, as a weight loss agent for obesity, and in the acute management of hyperglycemia [83].

Although apple seeds seem to be an interesting nutritional source, the presence of toxic cyanogenic glycosides critically limit their utilization as a food ingredient. The amygdalin content of seeds from 15 apple cultivars varied from 1 to 4 mg/g. These amygdalin contents could generate 0.06–0.2 mg cyanide equivalents per gram of apple seeds [87]. These values are relatively high, because acute cyanide toxicity can occur in humans at doses between 0.5 and 3.5 mg/kg body weight [88]. However, many processing techniques can reduce the cyanide content. Processing allows contact between cyanogenic glycosides and endogenous enzymes, which results in the hydrolytic breakdown of cyanogenic glycosides to hydrogen cyanide [87]. According to a murine study from Opyd et al. [81] a daily dose of 160 mg/kg body weight of apple seed meal for two weeks did not exert any observable adverse effects. The results showed increased protein digestibility as well as exerted beneficial effects on the intestinal tract, blood lipid profile, and antioxidant status of rats.

In conclusion, apple seeds are rich in nutrients, high-quality oil, and polyphenols, and can be a rich source of functional ingredients for health-promoting foods, feeds, nutraceuticals, and cosmetics. However, the contents of bioactivities vary significantly between various cultivars. In addition, for utilizing apple seeds, there is a risk concerning cyanogenic glucosides that must be considered, and the seeds must be processed in such a way that amygdalin is not physiologically toxic.

### 2.2. Grape (Vitis Labrusca and Vitis Vinifera)

Grapes are an important cash crop throughout the world. The berries stand out for their bright color, juicy flavor, and high nutrient content, and they are rich in vitamins, anthocyanins, carotenoids, and several antioxidant compounds, which are substances that can effectively remove free radicals and delay senility in the body [89,90].

As reported by The Plant List [91], the grape genus *Vitis* includes 68 species. Responsible for studying multiple genera, the Department of Agriculture (USDA) and the Natural Resources Conservation Service (NRCS) in the United States have classified the Vitis L., namely: *Vitis acerifolia* Raf. (mapleleaf grape), *Vitis aestivalis* Michx. (summer grape), *Vitis amurensis* Rupr. (Amur grape), *Vitis arizonica* Engelm. (canyon grape), *Vitis californica* Benth. (California wild grape), *Vitis cinerea* Engelm. ex Millardet (graybark grape), *Vitis girdiana* Munson (desert wild grape), *Vitis labrusca* L. (fox grape), *Vitis monticola* Buckley (sweet mountain grape), *Vitis mustangensis* Buckley (mustang grape), *Vitis* × *novae-angliae* Fernald (peregrine grape), *Vitis palmata* Vahl (catbird grape), *Vitis riparia* Michx (riverbank grape), *Vitis rotundifolia* Michx (muscadine), *Vitis rupestris* Scheele (sand grape), *Vitis shuttleworthii* House (calloose grape), *Vitis tiliifolia* Humb. and Bonpl. ex Schult. (West Indian Grape), *Vitis vinifera* L. (European or wine grape), *V. vinifera* L. ssp. *sylvestris* Hegi (wild grape), and *V. vulpina* L. (frost grape).

The basis of the phenolic composition of grapes and derived products is flavonoids, such as anthocyanins, flavones, and flavonols; stilbenes, such as trans-resveratrol; and phenolic acids, as gallic, vanillic, syringic, and caffeic [53]. Fiume et al. [92] suggested that amongst all the phenolic extractables in grapes, about 10% are present in the pulp, 28–35% are present in the peel, and 60–70% are present in the seeds. Several studies claim that there are several biological activities in grape phenolic compounds, both *in vitro* and *in vivo*, viz. anti-angiogenic, anti-proliferative, pro-apoptotic, antioxidant, and other functions [93,94,95,96,97]. Specific phenolic compounds extracted from grapevines studied by Hakimuddin et al., Singh et al., Schlachterman et al., and Singletary et al. [94,98,99,100] demonstrated anti-cancer and anti-metastatic activity, most especially when tested *in vivo* and *in vitro* against breast cancer cells. According to Yang et al. [101], the mechanism of action that could prevent tumor initiation may be related to the antioxidant, anti-inflammatory, and anti-proliferative activity of grape phenolic compounds.

Burin et al. [102] studied grape juice (Vitis vinifera cv. Cabernet Sauvignon), and determined using liquid chromatography the quantification of phenolic compounds as gallic acid: 82.6 mg/L; vanillic acid: 108.5 mg/L; ellagic acid: 543.4 mg/L; and *p*-coumaric 367.5 mg/L, with DPPH (1,1-diphenyl-2-picrylhydrazyl radical) and ABTS (2,2′-azino-bis(3-ethylbenzothiazoline-6-sulfonic acid) diammonium salt) antioxidant analyzes showing values of 191.1 and 213.5 µmol Trolox equivalents/100 mL, respectively. Granato et al. [103], also studying grape juices (*Vitis labrusca* cv. Bordeaux/Concord/Niagara/Isabel), determined by liquid chromatography the presence of 68 mg/L p-coumaric acid, 155 mg/L of chlorogenic acid, 108 mg/L of myricetin, 2.08 mg/L of *trans*-resveratrol, and 71 mg/L of malvidin 3,5-diglucoside, with 83.5% of transition metal-chelating ability (Fe^2+^) and 44.54% DPPH inhibition.

As stated in a review by Salehi et al. [104], V. vinifera wines contain a variety of phenolic compounds, such as hydroxybenzoic acids (e.g., gallate dimers), hydroxycinnamic acids (e.g., *trans*-caffeic acid), pyranoanthocyanins, flavonols (e.g., quercetin and myricetin), flavones (e.g., luteolin), and flavanols in their polymeric forms (e.g., procyanidins dimers). Tkacz et al. [105] document that the average amount of total polyphenolic compounds in white grape seeds (*Vitis vinifera* cv. Pinot Gris) exceeds by at least nine times the value found in the pulp and by more than five times what is found in the peel. The antioxidant activity was highest in seeds and lowest in pulp, as in a study by Yilmaz et al. [106].

Composed of skin, stems, and seeds, grape pomace represents the largest waste in the wine and juice industry [107,108]. The seeds represent about 5% of the fruit weight, generating approximately 40–50% of solid residues in the production process [109]. Grape seed oil has several applications, mainly in cosmetic formulations. It can contribute to nutraceutical effects due to its composition and antioxidant capacity [28]. Widely available in vegetables and fruits, flavan-3-ols represent the most common group of typical flavonoids of the human diet, and are present in higher concentrations in fruit seeds [110].

Consisting of high oil and polyphenols, grape seeds also contain oligosaccharides that display prebiotic activity [111,112]. According to Bordiga et al. [113], the oligosaccharides identified in grape seeds using chromatography can be considered, in some combinations, as a “functional ingredient” with potential prebiotic activity for *L. acidophilus* P18806, allowing improved growth during *in vitro* fermentation. Since grape seeds and skins are more valuable sources in compounds with high biological activity than fruit pulp, further studies to determine the biological properties with *in vivo* and *in vitro* testing of the morphological parts of the grape are very important for designing food products with high-sensory health-related quality. Moreover, seeds are an integral part of bagasse; thus, increasing their use in the food, pharmaceutical, and cosmetic industries would be economically justified.

Deolindo et al. [64] state that the hydroethanolic extracts of grape seed (*Vitis labrusca* cv. Bordeaux) have not only chemical antioxidant activity (based on DPPH analysis, where 3637 ± 4 mg AAE/100 g was obtained for the seeds and 1033 ± 10 mg AAE/100 g for the skin, extracted with a 60% *v/v* ethanolic solution), but also the potential to inhibit ACE-I (angiotensin-I-converting enzyme) activity *in vitro*. Salehi et al. [114] justify the inhibition of ACE-I activity to phenolic compounds in grape juice, peel, and seeds mainly due to the presence of (+)-catechin, (–)-epicatechin, (+)-gallocatechin and (+)-epigallocatechin, these substances being alternatives to the use of synthetic drugs to control or prevent hypertension.

Harbeoui et al. [65] confirmed the anti-inflammatory ability of the ethanol/water extract (80:20, *v/v*) of *Vitis vinifera* L. grape seeds by inhibiting the expression and production of different inflammatory markers. The authors also suggested that the different mechanisms involved in this anti-inflammatory activity could be due to the synergism between several phenolic compounds, as well as the specific structural elements of flavonoids (dominant fraction), constituting the primary determinants of anti-inflammatory activity.

Studies of the extract (ethanol/acidic water) of seed oils from cold-pressed fresh grapes, performed by Cecchi et al. [28], demonstrate *in vitro* inhibitory activity against PTP-1B (protein tyrosine phosphatase 1B enzyme), which is an overexpressed enzyme in type 2 diabetes, showing maximum inhibition values (98%) for *Vitis vinifera* cv. Sauvignon Blanc and minimal inhibition (40%) for *Vitis vinifera* cv. Cabernet Sauvignon. The authors assert an inhibitory activity exerted by the isolated phenolic fraction of these oils, and argue that these results urge for further studies, so that it is possible to confirm the action of grape seed oils and understand the mechanism behind them.

### 2.3. Pomegranate (Punica granatum)

The pomegranate (*Punica granatum*) is a fruit belonging to the *Punicaceae* family, which is native to Iran and is currently cultivated worldwide due to its great ability to adapt to various climatic conditions [43]. Pomegranates are mainly composed of bark, arils, and seeds, where residues can represent up to 50% of the total weight of the fruit [115]. Recent studies have sought to find methods to avoid wasting these residues by assessing the following: phenolic composition and antioxidant activity [42]; concentration of linoleic acid isomers [43]; *in vitro* and *in vivo* anthelmintic activity in relation to *Ascaridia galli* [116]; enrichment of fatty acid profile and reduced activity of desaturases in rat livers [117]; and decreased risk of non-communicable chronic diseases such as cancer, cardiovascular disease, diabetes, and obesity [44].

Ethanolic [41,42] and methanolic extracts [43] of pomegranate seeds were analysed on the presence of flavonoids, condensed tannins, tocochromanols, and carotenoids [44]. Ambigaipalan et al. [44] performed three different extractions of free phenolic compounds that were esterified and insoluble-bound. The authors demonstrated the abundance of phenolic compounds in pomegranate seeds and that different results can be reported when using different solvents. In the aforementioned study, 47 phenolic compounds were identified using HPLC-DAD-ESI-MS^n^, which were grouped into phenolic acids, flavonoids, proanthocyanidins, and anthocyanins. The following compounds were identified for the first time in pomegranate seeds: protocatechuic acid; vanillic acid; gallic acid; brevifolin carboxylic acid; *p*-hydroxybenzoic acid hexoside; *cis* and *trans*-caffeic acid hexoside; derivative of caffeic acid hexoside; vanillic acid hexoside; ferulic acid hexoside; catechin; quercetin hexoside; *cis* and *trans*-dihydrokaempferol hexoside; ellagic acid; ellagic acid pentoside; ellagic acid deoxyhexose; ellagic acid hexoside; valoneic acid bilactone; digalloyl hexoside; and galloyl-hexahydroxydiphenoyl hexoside.

Yoshime et al. [45] evaluated the conjugated α-linolenic acids (CLnAs) present in oil extracted by cold pressing pomegranate seeds, as well as bitter gourd seeds (*Momordica charantia* L.). These compounds were associated with anti-inflammatory and antioxidant properties. The fatty acid profile was characterized by gas chromatography and palmitic (16:0), stearic (18:0), oleic (18:1 ω-9), and linoleic acids (18:2 ω-6) were detected in both of the analysed oils. Punicic acid (PA; C18:3-9c11t13c) was the major fatty acid (>50%) present in pomegranate seed oil.

The antioxidant activity of pomegranate has been analysed in several studies. These included antioxidant activity by the DPPH method; antioxidant activity by the ABTS method; β-carotene by the bleaching method; antioxidant activity by electron paramagnetic resonance (EPR) spectrometry; metal-chelating ability; and oxygen radical absorbance capacity (ORAC) [41,43,44,45]. In these studies, pomegranate seeds showed antioxidant activity in all the methods that were used. Derakhshan et al. [42] evaluated the positive correlation between phenolic compound content and antioxidant activity using the β-carotene bleaching method. It was concluded that different methods should be employed to assess the types of antioxidant mechanism of action, such as single electron transfer, transition metal chelator capacity, and hydrogen atom transfer [118].

Biological assays with different protocols and experimental conditions are often used to evaluate natural products. In addition to identifying phenolic compounds, Ambigaipalan et al. [44] also studied the inhibitory effects of α-glucosidase and pancreatic lipase, the inhibition of peroxyl and hydroxyl radical-induced super stranded scission, and the inhibition of LDL cholesterol oxidation, confirming the effectiveness of the pomegranate seeds in the assays. Ferreira et al. [119] performed *in vivo* studies using mice and nanoemulsions of pomegranate seed oil for improving the photostability and antinociceptive effects of non-steroidal anti-inflammatory drugs. Zarepourfard et al. [120] performed *in vivo* assays on cloned goats to confirm that dietary supplementation with pomegranate seeds can improve sperm motility and viability after freeze–thawing, as well as maintaining developmental competence. Additionally, Białek et al. [117] performed dietary supplementation with pomegranate seed oil in Sprague–Dawley rats. The fatty acid content, lipid oxidation biomarker levels, and the activity of the main enzymes that catalyse lipid metabolism, confirmed that pomegranate seed oil is an alternative source of conjugated linoleic acids.

The use of pomegranate seeds for industrial purposes is already underway. A study by Van Nieuwenhove et al. [43] included pomegranate extracts as a component for the enrichment of conjugated linolenic acids in yoghurt. By using the DPPH method, there was a confirmed enrichment of the fatty acid profile, as well as an increase in antioxidant activity in the fermented products, while maintaining their nutritional, microbiological, and sensory characteristics. However, *in vitro* and *in vivo* evaluations of antimicrobial, cytotoxic, and antioxidant activity may be improved methods to study pomegranate seed extracts in greater detail.

### 2.4. Camu-Camu (Myrciaria Dubia H.B.K. McVaugh)

Camu-camu is a fruit species from the *Myrtaceae* family that naturally grows near rivers and lakes in the Amazon basin [121]. Pulp and peel extraction industries often use its fruits and seeds, which accounts for 50% of the total fruit weight and great loos if discarded [15].

The camu-camu seeds are a potentially rich source of phenolic compounds, while their functional and biological effects were previously reported. Studies such as those by Fidelis et al., Myoda et al., Azevêdo et al., and Maria et al. [15,46,47,48,49] have verified the high concentrations of phenolic compounds in the seeds, which are even higher than the concentrations from camu-camu pulp. Fidelis et al. [15] identified the presence of bioactive compounds by HPLC coupled to diode array detection. Fifteen phenolic compounds (phenolic acids, flavonoids, and trans-resveratrol) were identified in aqueous, ethanolic, and propanone extracts. There was a significant difference (*p* < 0.05) in the content of all the bioactive compounds among the three obtained extracts, except for 2-hydroxycinnamic acid. Differences in the solubility and polarity of the compounds may explain these differences, as varying polarity of solvents can result in a different compound being extracted.

Do Carmo et al. [49] performed five extractions with different proportions of water and EtOH for the *in vitro* evaluation of phenolic composition, antioxidant activity, cytotoxicity, and the inhibition of cisplatin-induced chromosomal aberrations. The extract with 50% of each solvent presented the highest concentrations of total phenolic compounds. The highest antioxidant capacity evaluated by three different methods (DPPH radical scavenging; ferric reducing–antioxidant power, FRAP; and Folin–Ciocalteu reducing capacity) also inhibited the growth of four cancer cell lines without cytotoxic effects on normal cells. In addition, the extract also presented a protective effect by decreasing the cisplatin-induced chromosomal break index by 37%.

Myoda et al. [46] analysed the antioxidant activity of camu-camu seeds through DPPH radical elimination and reduction power tests. They used five fractions of crude extract obtained with different proportions of water and methanol and verified the effectiveness of the extract on *in vitro* antioxidant activity. The aforementioned study evaluated the antimicrobial activity of the five fractions of the extract against *Staphylococcus aureus*, *Escherichia coli*, and *Saccharomyces cerevisiae* bacteria. Here, antibacterial action was only observed in relation to *Staphylococcus aureus* bacteria. A further study by Kaneshima et al. [122] isolated C-glycosidic ellagitannins in camu-camu seed extracts, which showed antioxidant action against DPPH and ABTS radicals.

The evaluation of antibacterial and cytotoxic effects was performed by Camere-Colarossi et al. [123], who compared methanolic extracts of camu-camu pulp and seeds. Both extracts showed antibacterial activities against *Streptococcus mutans* (ATCC 25175) and *Streptococcus sanguinis* (ATCC 10556) bacteria. However, the seed extract presented the largest inhibitory halos, while the extracts showed no cytotoxic effects at the concentrations used.

Regarding *in vivo* studies, Yazawa et al. [124] studied the methanolic extracts of camu-camu seeds, and found anti-inflammatory action in carrageenan-induced paw oedema in murine models. Studies of camu-camu pulp are more frequently found in the literature [125,126,127,128,129]. However, it was stressed to use the waste that is generated while obtaining the pulp. *In vitro* and *in vivo* research with camu-camu seeds should be further developed, because this residue is recognized as a source of high concentrations of bioactive compounds and antioxidants.

### 2.5. Plums (Prunus *sp.*)

Plums (*Prunus* sp.) are among the most popular processed fruits. The global production of plums has been increasing and reached 11.8 million tons in 2017 [12]. Most often, plums are processed into dry fruits, jams, and juices, which generate tons of seeds as by-products. Thus, utilization of the seeds is of growing interest due to environmental and economic aspects.

The oil content of plum seeds varies from 23% (*w/w*) to 53% (*w/w*) per dry weight basis, depending mainly on the variety [73]. The dominant fatty acids in plum seed oil are oleic and linolenic acids; oleic acid constitutes 46–71% of the oil, and linoleic acid constitutes 23–45% of the oil, depending on the variety [73]. Based on the high content of unsaturated fatty acids, plum seeds have potential to lower LDL cholesterol and prevent cardiovascular diseases [79]. The oil is also rich in bioactive compounds, such as carotenoids, tocopherols, tocotrienols, phytosterols, and squalene [73]. The content of tocochromanols in 100 g of the oil is reported to be up to 209 mg, the content of total carotenoids is reported to be up to 3 mg, the content of phytosterols is reported to be up to 1579 mg, and the content of squalene is reported to be up to 80 mg [73]. Although the content of the bioactives varies greatly between the varieties, β-sitosterol and γ-tocopherol remain the main lipophilic compounds in plum seed oils. The contents of β-sitosterol constitutes are 209–1259 mg/100 g of oil and γ-tocopherol constitutes are 61 and 182 mg/100 g of oil [73].

Phytosterols, such as plant stanols and sterols, are steroid compounds that have a significant impact on human health. For example, phytosterols can reduce the levels of cholesterol in blood serum [130]. For example, tocochromanols include tocopherol and tocotrienol, which are active forms of vitamin E. They have important functions in human nutrition, and they possess various physiological and biological activities, such as the prevention of cardiovascular diseases and diabetes (e.g., [131]). Carotenoids are natural isoprenoid pigments with various biological and physiological functions, such as antioxidant and antitumor activities (e.g., [132]). One of the most common carotenoids in the plants is β-carotene, which exhibits pro-vitamin A activity. Squalene (2,6,10,15,19,23-hexamethyl-2,6,10,14,18,20-tetracosahexane) is a biochemical precursor of vitamin D, cholesterol, and steroid hormones, and thus is of high importance for human health [133].

In addition to the healthy fatty acids, lipophilic bioactives, and polyphenols, plum seeds have been characterized as a source of functional proteins and bioactive peptides. The protein content of plum seeds is up to 40% on a dry matter basis [134]. High-intensity ultrasound treatment has been applied for extracting the proteins [134,135]. The produced protein concentrates have been shown to possess good solubility, emulsifying, and foaming properties, as well as gel and film formation capacity. These functional properties indicate that the plum seed protein concentrates could meet the complex requirements of manufactured food products. For example, excellent emulsifying and gel-forming properties are needed in sausage products and emulsifying and film-forming capacities enable the production of emulsion-based edible films. In addition, proteins can be used for the production of biologically active peptides. This far, plum seed protein-derived peptides have shown antioxidant and antihypertensive (angiotensin I converting enzyme inhibitory) properties *in vitro* [134,136]. Bioactive peptides have been produced by hydrolyzing the plum seed proteins with proteolytic enzymes. Among the tested enzymes, alcalase most efficiently released the antioxidant and potentially antihypertensive peptides. The peptide sequences IYSPH, IYTPH, IFSPR, and VAIP have been reported to relate to the potential antihypertensive activity [136].

In summary, plum seeds are rich sources of nutrients, especially oils and proteins, and a wide variety of natural bioactive compounds. Based on these compounds, plum seeds have potential for exploitation in health-enhancing functional foods, nutraceuticals, extending the shelf life and active ingredients in food and cosmetic products. However, it needs to be taken into account that the concentration of the bioactive compounds varies significantly between the different varieties, and also, the seeds contain toxic cyanogenic glycosides, which need to be diminished during processing.

### 2.6. Jabuticaba (Plinia jaboticaba and Plinia cauliflora *Mart. Kausel*)

Jabuticabeira belongs to the *Myrtaceae* family, and it is a native species of Brazil with great economic importance. It is produced mainly in the states of Minas Gerais, Espirito Santo, Rio de Janeiro, São Paulo, and Paraná [137]. Due to its high water and sugar content, jabuticaba is highly perishable, and is not widely consumed in its fresh form. On the other hand, its pulp is usually extracted to make jams, wines, liqueurs, and vinegars [54]. According to Jorge et al. [138], the residues generated in the extraction of this fruit are seeds and peel, which represent approximately 50% of the fruit. The purposeful processing of these residues would reduce the production of organic wastes and add value to the food products, because they are a rich source of bioactive compounds, e.g., dietary fiber, minerals, and phenolic compounds [139].

Extraction is fundamental to obtain accurate results in relation to the studied matrix. To evaluate the phenolic compound content in jabuticaba seeds, Paludo et al. [53] performed optimized extraction using three solvents (water, EtOH, and methanol) and compared combinations of these solvents. The EtOH:water composition (60:40 *v/v*) had the highest extraction efficiency and proved the presence of high levels of phenolic compounds in the jabuticaba seeds. Inada et al. [54] evaluated the technological potential of the fractions (peel, seeds, and pulp) of jabuticaba and determined the antioxidant capacity and chemical composition. In the identification of phenolic compounds, soluble and insoluble fractions were extracted, and gallic acid was found to be the main compound present in jabuticaba seeds. The fractions showed *in vitro* antioxidant capacity in the methods used (Folin–Ciocalteu; ferric-reducing ability, Trolox equivalent antioxidant capacity, and oxygen radical antioxidant capacity). A positive correlation was observed between total phenolic compounds and antioxidant capacity, regardless of the assay, indicating that these bioactive compounds are effective in antioxidant action.

Similarly, Hacke et al. [55] performed the extraction and chemical characterization of antioxidant and antimicrobial compounds from jabuticaba seeds. The effects of three solvents (water, ethyl alcohol, and propanone) were analysed, and the binary combination of water and propanone (60:40 *v/v*) obtained the best results regarding phenolic content and antioxidant activity using the DPPH method After optimization, the crude extract was purified, and ellagic acid and ellagitannin compounds were identified using electrospray ionization coupled with tandem mass spectrometry (ESI-MS/MS). Alezandro et al. [56] evaluated jabuticaba fractions and also reported the presence of ellagic acid derivatives in jabuticaba seeds. In this same study, the methanolic extract of jabuticaba seeds presented higher antioxidant capacity when compared to the skin and pulp extracts, regardless of the method used (Folin–Ciocalteu reducing capacity, DPPH scavenging ability, or ferric-reducing ability). This study demonstrates how important it is to consume the whole fruit, because each fraction has its own particular importance and helps to maintain health.

Jorge et al. and Lima et al. [138,140] evaluated the profile of fatty acids and organic acids in jabuticaba seeds, respectively. Regarding fatty acids, polyunsaturated fatty acid concentrations were obtained, with a predominance of linoleic acid and α-linolenic acid. The organic acids that were detected included citric acid, succinic acid, malic acid, oxalic acid, and acetic acid. Such compounds are essential in maintaining health, because they contain beneficial properties for the body such as antioxidant, antimicrobial, and anti-inflammatory capacity.

In a practical use of jabuticaba seeds, Baldin et al. [139] evaluated the antioxidant and antimicrobial activity of the microencapsulated aqueous extract of jabuticaba residues (bark and seeds) used as dyes in the manufacture of mortadella. The addition of the extract provided better sensory acceptance, and did not interfere with the chemical composition, lipid oxidation, and bacterial development. According to the authors, the addition of natural extracts is promising and represents great potential for application during the manufacture of products enriched with natural antioxidants.

Jabuticaba peel has been identified as a potential source of bioactive compounds with biological activities. *In vivo* studies were performed by Batista et al. [141], who obtained an improvement in the triglyceride excretion and hepatic lipid peroxidation in rats fed a high-fat diet enriched with lyophilized jabuticaba peel. Palozi et al. [142] showed that the oral use of ethanolic extracts of jabuticaba peel in rabbits did not cause significant changes in respiratory, cardiovascular, and central nervous system functions, ensuring pharmacological safety for the use of this residue. However, biological studies of jabuticaba seeds are scarce, and further research is required to assure the complete safety of these potential sources of bioactive compounds. Hacke et al. [55] evaluated the protective effects of jabuticaba seed extract using *in vivo* micromolecular assays on murine bone marrow cells combined with cyclophosphamide. Ellagitannins (castalagin, vescalagin, and pedunculagin) were the main compounds isolated from jabuticaba seed extracts, which protected DNA from damage and accelerated DNA reparation.

Future research regarding jabuticaba seeds, particularly in relation to *in vitro* antioxidant, antimicrobial, and antiproliferative activities, as well as *in vivo* analysis, may provide more specific information on how to best utilize these residues.

### 2.7. Avocado (Persea americana *Mill.*)

*Persea americana* (Lauraceae), commonly known as avocado, is native to Mexico and Central America, and can be widely found throughout tropical countries. Most of avocado’s chemicals and bioactivity studies are focused on pulp, but little is known about the seed. Extensive research on this part of the fruit may be of great interest due to its anti-inflammatory properties (decreasing the generation of interleukin-6 pro-inflammatory mediators, IL-6; and prostaglandin-E2, PGE2; anti-cancer, antioxidant, and antihypertensive factors). Numerous chemical characterizations have shown a large number of polyphenols, such as catechins, procyanidins and other tannins, flavonoids, triterpenes, and unsaturated fatty acids in avocado seeds [143,144].

Using gas chromatography (GC) for the lipid extraction of avocado seeds, Alkhalf et al. [51] found linoleic (39.43%), oleic (25.30%), palmitic (20.05%), palmitoleic (3.66%), stearic (3.36%), and α-linoleic acids (2.62%). According to Alkhalf et al. [51], the extraction (chloroform/methanol) showed antioxidant activity using two electron transfer methods (ABTS and DPPH) at a concentration of 2 × 10^−4^ g/mL (69.73% and 36.64% inhibition, respectively) as compared to Trolox (6-hydroxy-2,5,7,8-tetramethylchroman-2-carboxylic acid) and BHT (2,6-bis(1,1-dimethylethyl)-4-methylphenol). The lipid extract (chloroform/methanol) of the seeds recorded significantly higher activity than that of the fruit. Through an *in vitro* experiment on human red blood cells, it was also revealed that both the pulp and seeds have significant dose-dependent anti-inflammatory potential compared to a control standard (membrane stabilization method). Still, according to Alkhalf et al. [51], the results show the antiproliferative activity of avocado seed and pulp lipid extractions in hepatocellular carcinoma (HepG2) and colon cancer (HCT116) cell lines.

*In vivo* studies performed by Athaydes et al. [61] demonstrated that avocado seed extracts with ethyl acetate have gastroprotective activity and can be used as an adjunctive treatment for indomethacin-induced gastric mucosal injury. Uchenna et al. [145] have shown promising component values in avocado seeds as a source of energy, protein, and bioactive phytochemicals to stimulate growth and metabolism. Additionally, the authors reported that the inclusion of avocado seeds in diets has also lowered cholesterol levels and suppressed hyperglycemia in rats.

Studies on avocado seed pigmentation conducted by Hatzakis et al. [146] demonstrated that the seed has perseorangan as the most abundant component, which may contribute to its final coloration. The authors stated that further research is needed to characterize the usefulness of this compound as a food coloring additive and to identify its biosynthetic precursors and potential natural derivatives. Likewise, toxicological studies should be performed on cells and mice to ascertain its safety. *In vitro* and *in vivo* studies of the extract from avocado seeds could be an alternative to better understand its biological activities and encourage its use as an ingredient in the cosmetic, pharmaceutical, and food industries.

### 2.8. Passion Fruit (Passiflora edulis *Sims*)

*Passiflora*, originally from the tropical and warm climates of South America, is the largest genus in the *Passifloraceae* family, containing about 500 different species. Due to their pleasant natural and intense bittersweet flavor, *Passiflora* fruits, popularly known as passion fruit, are much appreciated in the world. In the food industry, its juice is the main source of nutritional properties, mainly due to the presence of many phytochemical constituents such as phenolic compounds. Although hundreds of *Passiflora* species can be found worldwide, only a few are identified as edible. In addition, these fruits contribute to some beneficial health properties, such as antioxidant, anti-inflammatory, antipyretic, analgesic, sedative, and hypotensive activities [67,147,148,149]. *Passiflora* fruits are also rich in minerals (calcium and phosphorus), retinol, ascorbic acid, thiamine, riboflavin, and niacin [150].

*In vitro* studies using enzymes as a substrate show that phenolic and flavonoid compounds can inactivate α-amylase, α-glucosidase, and lipase through non-specific enzyme binding [151]. *Passiflora* species, such as *P. edulis (flavicarpa), P. alata, P. incarnata, P. mollissima, P. tripartita, P. ligularis*, and *P. quadrangularis* are widely cultivated and hold several biologically active secondary metabolites, including phenolic compounds.

*C*-Glycosylated derivatives of apigenin and luteolin, vitexin, isovitexin, orientin, schaftoside, 2-*O*-rhamnoside, luteolin-7-*O*-(2-rhamnoside), quercetin 3-β-glucoside, and iso-scoparin-2-*O*-glucosides are some new functional compounds of the *Passiflora* species [67,152,153]. According to Shanmugam et al. [149], protocatechuic acid is the main component found in *P. subpeltata* fruit pulp, quantified as 115.43 ηg/g, followed by ferulic acid (55.76 ηg/g), vanillic acid (46, 01 ηg/g), (–)-epicatechin (22.45 ηg/g), and *p*-coumaric acid (20.54 ηg/g). In addition, three compounds were found: cinnamic acid (9.08 ηg/g), eriodictyol (8.02 ηg/g), and quercetin-3-glucoside (3.04 ηg/g). Through DPPH analysis, the value of half of the maximum inhibitory concentration (IC_50_) was (5667 μg/mL) of fresh pulp, which is very close to standard rutin (5670 μg/mL) and BHT (6380 μg/mL) values. The maximum values found in fresh pulp relate to its antioxidant capacity, which eliminates free radicals by hydrogen donation, as defined by a previously reported hypothesis [154]. Rotta et al. [70], studying *Passiflora* species using liquid chromatography and the QuEChERS extraction method (quick, easy, cheap, effective, rugged, and safe), state that fruit pulp is an important source of phenolic compounds. In their studies with *Passiflora* pulp extract (acetonitrile), they determined phenolic compounds (quercetin, rutin, 4-hydroxybenzoic, chlorogenic, ferulic, vanillic, caffeic, *trans*-cinnamic, and *p*-coumaric acids). They also state that *P. edulis* showed the highest abundance of vanillic acid (426 ± 29 40 µg/kg) and quercetin (416 ± 6 µg/kg), whereas in *P. alata* and *P. ligularis* species, rutin (289 ± 6 41 µg / kg) and caffeic acid (64 ± 2 µg/kg), respectively, were found at the highest levels. Through DPPH and ABTS analyses, the authors concluded that all the extracts studied present antioxidant activity and phenolic content.

Studying tropical fruit by-products water extracts (mix of shell and seed in powder form), Albuquerque et al. [66] assert their potential to act or be a source of antioxidant dietary fiber. The passion fruit extract evaluated in the study is capable of acting as a prebiotic with antioxidant activity when analyzed through ORAC (oxygen radical antioxidant capacity) and DPPH. Using various types of extractions including maceration, (MAC), ultrasound-assisted leaching (UE), supercritical fluid extraction (SFE), and solvents (hexane, ethanol, ethanol/water and ethyl acetate), Oliveira et al. [69] demonstrated the antimicrobial activity of passion fruit (*P. edulis* sp.) seed in *E. coli* and *L. innocua*, presenting minimum inhibitory concentration (MIC) values of 8.0 mg/mL in the combination of maceration extraction (ethanol/water) and UE (ethanol/water); and 8.0 mg/mL in the combination of supercritical fluid extraction–150/300 bar 40 °C and 4.0 mg/mL 150 bar 50 °C, respectively. Lam and Ng [155], studying a new dimeric protein from passion fruit seeds, *P. edulis* (passiflin), determined specific antifungal activity against *Rhizoctonia solani* with an IC_50_ of 16 µM and a potent inhibitory action on breast cancer cells (MCF-7) with an IC_50_ of 15 µM.

Lourith and Kanlayavattanakul [156], studying the ethanolic extract of *P. edulis* seeds, state that the processing residue of passion fruit juice (seed) is a source of functional compounds, and the antioxidant fraction, determined by DPPH, FRAP, and ABTS analyses, works as a sunscreen with an effective protection against UV rays, as well as a skin-whitening agent. The sun protection capability of the extracts was confirmed by the fact that it contains quercetin and rosmarinic acid in its composition, which have potential action against photooxidative damage. Thus, this eco-friendly antioxidant is considered safe and effective for health-beneficial uses, and therefore, passion fruit seed can be used as an ingredient in the development of cosmetics, especially anti-aging and anti-wrinkle products, which are effective in protecting against UV, combating photoaging.

Malacrida and Jorge [157] confirmed through the study of passion fruit seed methanolic extract that the antioxidant amount present in the seed can serve as a dietary source of natural antioxidants, helping to prevent diseases by combating free radicals *in vivo* or as a food additive, increasing the stability and quality of food products. This antioxidant activity is attributed to phenolic compounds (linoleic, oleic, palmitic, and stearic) and to the presence of δ-tocopherol and γ-tocopherol (natural antioxidants). The authors further state that oil extraction from passion fruit seeds could add value to products, which is generally discarded as waste. The seed can also be used as a raw material in different industries, including food, detergents, cosmetics, supplements, vitamins, and biodiesel.

The results obtained by Macagnan et al. [158] suggest that apple, orange, and passion fruit by-products positively influence lipids and glucose metabolism, without negative effects *in vivo*, not affecting the growth of mice studied during the experiment period. By-products promoted a significant reduction in triglyceride and hepatic cholesterol levels and exerted important effects on postprandial glucose control. Furthermore, the authors found that these fiber sources are important for intestinal health without affecting the regularity of intestinal transit, demonstrating that apple pomace, orange pomace, and passion fruit peel can be considered as fonts of dietary fiber with relevant functional properties to health promotion and protection.

### 2.9. Berry Seeds 

Most of the research published so far has been focused on the berry seeds from different species of *Rubus* (including blackberry, *R*. *ulmifolius*, *R*. *fruticosus*; raspberry, *R*. *idaeus*, *R*. *occidentalis*; and cloudberry, *R*. *chamaemorus*) and *Vaccinium* (blueberry, *V*. *corymbosum*; bilberry, *V*. *myrtillus*; and cranberry, *V*. *oxycoccus*, *V*. *macrocarpon*, *V*. *oxycoccos*). The seeds of sea buckthorn (*Hippophae rhamnoides*), strawberry (*Fragaria* × *ananassa*), and blackcurrant (*Ribes nigrum*) have been investigated. The major bioactive compounds and bioactivities of berry seeds are summarized in Table 2 [19,159,160,161,162,163,164,165,166,167,168,169,170].

The bioactive compounds found in berry seeds mainly are phenolic compounds, fatty acids, and tocopherols. Previous studies shown in Table 2 showed a large deviation in the total content of phenolic compounds in each berry species. It is difficult to compare the abundance of phenolics among different berry seeds, since the values are influenced by many factors, such as cultivars of berries, cultivation condition, extraction solvents, and analytical methods. The primary phenolic compounds in seeds are dependent on the berry species. Ayoub et al. [19] compared the phenolic profiles of seed meals of blackberry, raspberry (black), and blueberry.

Blackberry seeds contained the highest amounts of total phenolics (13 mg gallic acid equivalents - GAE/g defatted seed meals), followed raspberry (7 mg GAE/g) and blueberry (2 mg GAE/g). Based on the content obtained by HPLC, the phenolic compounds in these three seeds mainly are phenolic acids, proanthocyanidins, flavan-3-ols, flavonols, and anthocyanins; representing as free, esterified, and insoluble-bound forms. Gallic acid and its derivatives were the main phenolic acids in all berry seed meals. For flavonoids, B-type procyanidin dimers were the dominant compounds in blueberry seeds. However, quercetin, together with its glycosides, accounted for over 50% of the total flavonoids in the seeds of both blackberry and raspberry. van Hoed and co-workers investigated phenolic compounds in the seed oils of raspberry (red), blackberry, cranberry, blueberry, and strawberry by using HPLC-DAD [167]. The total amount of phenolics (mainly as phenolic acids) in cold-pressed berry seed oils varied from 0.1 (blackberry) to 15.8 mg/g of oil (strawberry). *p*-Coumaric acid formed the major phenolic compounds in the seed oils of cranberry, while 4-(2-hydroxyethyl)phenol was dominant in both strawberry and raspberry seed oils. Other compounds, such as homovanillic acid, vanillic acid, vanillin, and protocatechuic acid were quantified in studied oil samples. Ellagitannins were abundant in the seeds of cloudberries and blackberries [171,172]. The primary isomers included pedunculagin, casuarictin/potentillin, castalagin/vescalagin, lambertianin A/sanguiin H-6, lambertianin C, and lambertianin D. The wealth of these isomers varied in the different cultivars of berries [166]. As a large group of phenolics, lignans were also detected form the seeds of cloudberry, blackberry, cranberry, bilberry, raspberry, sea buckthorn, and blackcurrant. The total content ranged from 0.02 (blackcurrant) to 0.4 mg/g of dry seeds (cloudberry) [173].

Previous research suggested that fatty acids in berry seeds were present mostly as polyunsaturated fatty acids. They accounted for 83% in raspberry seeds [160], 65–78% in strawberry seeds [159,160], 78–79% in blackcurrant seeds [159,170], 75% in cranberry seeds [159], and 64% in bilberry seeds [159]. Although the concentrations varied among berry species, the dominant fatty acids were linoleic acid (C18:2, n-6, 12–62%), α-linolenic acid (C18:3, n-3, 4–44%), and oleic acid (C18:1, n-9, 4–45%) [159,160,163,164,165,170,174,175]. The n-6/n-3 ratio was close to 1 in the seed oils of elderberry (*Sambucus nigra*), strawberry, cranberry, bilberry, and raspberry, while the value ranged from 2.7 to 3.6 in blackberry and blackcurrant seeds [159,160,163,164,165,170]. Both tocopherols and tocotrienols were detected in berry seeds. The good sources were the seeds of raspberry (44–420 mg/100 g oil, mainly as γ-tocopherol), cloudberry (270, α-tocopherol and γ-tocopherol), cranberry (114–195, γ-tocotrienol), blackberry (164, γ-tocopherol), sea buckthorn (100–200, α-tocopherol and γ-tocopherol), lingonberry (*Vaccinium vitis-idaea*, 130, γ-tocotrienol), elderberry (122, γ-tocotrienol), and blackcurrants (106–110, α-tocopherol and γ-tocopherol) [159,160,163,167,174].

The antioxidant activity of berry seeds has been studied extensively [19,159,160,161,162,163,164,165,167,168,169,170]. Most of the studied seed oils or seed extracts have shown strong capacities of directly scavenging free radicals, chelating metal cations, or inhibiting the oxidation (or DNA damage) caused by free radicals. Yet, the compounds responsible for anti-oxidative effects have not been determined, which is likely due to the large chemical diversity that was found in the extracts. The related conclusions have been drawn only based on the correlation between antioxidant results and the concentration of chemical components. Furthermore, it is difficult to rank berry seed extracts based on their antioxidant effects due to different extraction solvents applied. For example, Helbig and co-workers evaluated both the hexane and water extracts of several seed press residues using the trolox equivalent antioxidant capacity - TEAC method [159]. The results showed that the antioxidant capacity of both hexane and water extracts decreased in the order of elderberry > blackcurrant > cranberry > bilberry. Similarity might be caused by the presence of different bioactive compounds in extracts. Additionally, considering the nature of free radicals, the inhibitory effects of certain berry seeds might differ in various antioxidant assays. Yang et al. reported the peroxyl radical scavenging capacities of supercritical CO_2_ extracted berry seed oils [163]. The activity was positively correlated with the content of tocopherols and tocotrienols in the seed oils. Among the seed oils studied, raspberry seed oils exhibited the best activity against peroxyl radicals, but the lowest efficacy of inhibiting lipid oxidation compared to seed oils from the other species of the *Rubus* and *Vaccinium* families. In contrast, bilberry seed oil was a potent inhibitor against lipid oxidation, yet weakened with scavenging peroxyl radicals [163]. In the same research, the authors reported the protective effects of sea buckthorn seed oil against oxidative damages of DNA.

Regarding other bioactivities of berry seed extracts, Basu et al. [176] confirmed that that supercritical CO_2_-extracted sea buckthorn seed oil had a significant anti-atherogenic activity when administrated to normal or hypercholesterolemic rabbits. The ethanolic extracts of sea buckthorn seeds strongly inhibited the growth of *Enterecoccus durans* (68%), *Candida albicans* (68%), and *Bacillus cereus* (64%). This was attributed to the high levels of total phenolic compounds, which were represented primarily with condensed tannins [161]. Puupponen-Pimiä et al. [169] evaluated two fractions of acetone extracts of cloudberry seeds in liquid cultures of selected microbial strains. Both fine and seed coarse seed fractions showed strong antibacterial efficacy on the growth of *Staphylococcus aureus* and *Escherichia coli*, whereas inhibition was observed on *C. albicans* and *Saccharomyces cerevisiae*. It was speculated that high contents of casuarictin/potentillin and sanguiin H2 isomers in the fractions might be responsible for the strong antimicrobial activity [171]. In the same study, the anti-inflammatory abilities of cloudberry seeds were estimated in a dose-dependent manner and caused a significant decrease on pro-inflammatory factors (bacterial lipopolysaccharide induced nitric oxide, NO, IL-6, and inducible nitric oxide synthase, iNOS). As compared to the seed fine faction, the coarse counterpart had the stronger anti-inflammatory effects at the concentration of 1–3 µg/mL, which was associated with the presence of quercetin derivatives [171]. The methanolic extracts of seeds of blackberry and elderberry were tested against bacterial lipopolysaccharide-induced NO and CCL20 in macrophages. The results revealed that blackberry seed extracts had a strong anti-inflammatory property, inhibiting 60% of NO and over 90% of CCL20 production at the dose level of 50 µg/mL. Nevertheless, no inhibitory effect was detected in elderberry seed extracts [165].

The clinical evidence on the health-promoting effects of berry seeds has been summarized by Yang and Kortesniemi [177]. Dietary supplementations with berry seed oils have shown positive impacts on atopic dermatitis [178], plasma lipid profiles [178,179], and platelet aggregation [180,181]. The beneficial effects were due to the high content of polyunsaturated fatty acids with favorable ratios of ω6/ω3 that were close to the recommendation given by the global experts. However, other lipophilic components such as phytosterols, tocopherols, and tocotrienols may also play an important role. [101,163,181] reported that the supplementation with 2 g of sea buckthorn oil (extracted from both seed and pulp) for three months has been shown to have a positive effect on human subjects between 20 and 70 years old suffering from dry eye symptoms [179]. Moreover, Linnamaa et al. [182] suggested that the prevalence of atopic dermatitis in infants could be reduced by dietary intervention using blackcurrant seed oil, when administered to both women during pregnancy and lactation, and to the babies after weaning.

## 3. Extraction Technologies of Water-Soluble and Lipophilic Bioactive Compounds

The extraction of bioactive compounds has a long tradition using conventional solvent extraction techniques based on different organic solvents. However, the demand for improving the extraction yield of the target bioactive compounds from plant matrices and the pressure on the environmental friendliness of the production process indicated the need for the development of novel extraction techniques that are based on benign pre-extraction technology by the implementation of enzymes [183]. Marathe et al. [184] described the effectiveness of the enzymatic hydrolysis in the reduction of cellular wall thickness to ensure the better passage of the solvent toward compounds that may be bound in the wall. This way, the extraction will eventually lead to higher yields, more efficiency, a reduction in the processing time and the solvent used, and at the end, a prospective technological solution that is sustainability and commercially viable.

Different enzymes can be used to facilitate the extraction of bioactive compounds, e.g., phenolics from grape seed as an example. Fernández et al. [185] compared the effectiveness of pectinases, cellulases, and tannases, and the blend of these enzymes. All the studied enzymes increased the total phenolic content in the extracts (versus controls) with a 1.18–1.34-fold increased yield. Comprehensive collection on the findings for enzymatic aids in processing for the extraction of polyphenols was recently published by Gligor et al. [186]. This review gives information on the use of hemicellulases and lignanases for the hydrolysis and oxidizing of cellular walls. However, González-García et al. [136] describe that the use of lignanases is not viable for food industrial applications, due to the formation of low-molecular reactive compounds in the oxidation process. The authors reported that although there are positive outcomes regarding the efficiency and environmental friendliness of enzyme-assisted extractions, the high costs and low popularity slows down their prevalence for food industrial applications.

Recently, the combination of these enzymes with microwave, ultrasound, supercritical fluid extraction, or high-pressure extraction was described in order to overcome the drawbacks mentioned above. The target compounds that strongly bind to the cell walls usually will not be fully released with enzymatic digestion by polysaccharide-degrading enzymes or hemicellulases [187]. It was reported that approximately 24% of the total phenolics are bound in the food matrices with polysaccharides (e.g., hemicellulose, cellulose, and pectin), and with certain biomolecules, as proteins. From this point of view, enzymatic utilization of a different nature can significantly enhance the recovery of the phenolics. Pap et al. [188] showed that the enzymatic depectinization of the blackcurrant juice by pectinase significantly increased the amount of available flavonols, but a similar observation for anthocyanins were not observed. Fernandes and Carvalho [189] furthermore explained that the treatment with cellulases, hemicellulases, and pectines resulted in the formation of smaller oligosaccharide chains and the liberation of compounds, such as oils and proteins.

Ultrasound techniques are based on the acoustic cavitation in which small vapor-filled bubbles are generated. These bubbles will eventually explode and result in high pressure and temperature in the cell walls [190]. Ultrasound waves that are exploited for the treatments of different food materials are generally over 20 kHz [191]. The application of ultrasound extraction (US) for polyphenols from different fruit seeds has already been covered in the literature. Segovia et al. [62] studied the batch and continuous US for recovering polyphenols from avocado seeds. These authors also modeled the extraction process and described that the film theory and Fick’s law models could sufficiently describe the US process, for both batch and continuous phase. Such a mathematical modeling provides understanding on the proceeding of the extraction.

Da Porto et al. [192] investigated the extraction of oils and polyphenols in US from grape seeds and compared the results with conventional extraction. The results indicated that the removal of oil was almost as efficient with US as with Soxhlet when a power of 150 W for 30 min was used. Comparing the polyphenolic extraction from grape seed by maceration versus US, these authors reported that the preliminary treatment with US increased the yield of polyphenols in maceration. However, a significant decrease of polyphenolic content was observed with double US treatments, when both the oil removal and the polyphenolic extraction were done by the US. Since polyphenolic compounds are naturally heat sensitive, this observation can be attributed to the significantly higher temperatures and pressures in the US hot spots [193] and consequential breakdown of the polyphenols.

Opposite to ultrasounds, microwaves are non-ionizing radiations at frequencies between 300 MHz to 300 GHz [194]. Considering the mechanism of microwave action, they are different in nature in a way that heating, and the extent of the absorbed heat will depend on the ability of polar solvents to absorb the microwave energy. Ionic conduction and dipole rotation of the solvent will result with heating, which is selective in nature, and opposite to conventional extractions where generally, the vessel is heated, and the solvent absorbs the heat through it. Li et al. [195] reported the effectiveness of the microwave-assisted extraction (MAE) of polyphenols from grape seeds. The authors optimized the MAE of the grape seeds in a five-level, three independent variable central composite rotatable design, and concluded that the yield of polyphenols in MAE extracts were comparable to that of US extraction and conventional extraction. However, a significant benefit was the much shorter time that was required for the extraction. The length of the process is always a crucial factor, since some of the compounds, especially heat-sensitive ones, may start to degrade during the course of the processing. On the other hand, Bucić-Kojić et al. [196] developed a statistical model for the description of the kinetics of grape seed phenolics depending on the temperature. The understanding and the implementation of such modeling is an extremely useful tool in process scale-up and industrial application as well.

Pressurized hot water extraction also plays an important role in novel extraction techniques. This extraction technology is gaining more interest for the production of bioactive compounds. Here, the pressure is adjusted to keep the water in its liquid state, and the temperature is between the boiling and critical point [197]. This technology benefits from the changes of the dielectric constant of water, as increasing the temperature up to 200–350 °C will decrease the dielectric constant of the water and provide similar polarities to those that methanol, EtOH, or acetone have at room temperature [198]. The hot water is usually recirculated, or the process can be also multi-staged. The complexity of the technology increases when matrices with multiple compounds are used in real applications, since the operation parameters must be well manipulated in order to extract the desired compound without the other contaminants. However, it is important to avoid undesirable changes of the target compounds that could deteriorate during the extraction. Plaza and Marina [199] published a comprehensive review on the potential application of the pressurized hot water extraction. The application for the extraction of phenolic compounds, diterpenes, triperpenes, polysaccharides, and proteins from different plant materials, peels, leaves, seeds, barks, and wastes were covered, and a positive trend for their future importance was indicated by this technology. Beside, several authors reported the results of the study on the extraction of polyphenolic compounds [198,200] from grape peels and pomegranate seed residues [201] for anthocyanins from grape pomace [202].

In supercritical fluid extraction, the most commonly used solvent is carbon dioxide, which due to its low critical temperature allows the extraction of thermally sensitive compounds [184,203]. Still, the challenge is related to the high cost of processing as compared to a low yield of extracted compounds. Nonetheless, the nature of the CO_2_ as a solvent enables the extraction of compounds with non-polar character. However, when used alone in the extraction, it is inadequate for the extraction of highly polar compounds, e.g., polyphenols being one of them [204]. Due to this, for the most polyphenolic recovery from plant seeds, the CO_2_ is combined with other solvents, (e.g., ethanol) for potential applications in food production. Castro-Vargas et al. [129] extracted bioactives from guava seeds with supercritical CO_2_ extraction combined with EtOH, and found a correlation between the applied pressure and temperature on the antioxidant activity of the extracts.

Yilmaz et al. [203] pointed out that the challenge with the valorization of the grape seeds is the residual oil obtained along with the phenolic compounds. Hexane is commonly used for the conventional extraction of oil, but it needs additional evaporation step(s) to remove the residual solvent after the defatting. The authors reported that with the co-addition of ethanol in the supercritical extraction, with a suitable adjustment of pressure and temperature, proanthocyanidins were successfully recovered from the grape seeds. Da Porto and Natolino [205] used response surface optimization to estimate the optimum process parameters for the extraction of polyphenols from white grape seeds in a two-stage supercritical CO_2_ extraction. The optimization was carried out by studying the effects of pressure, the amount of ethanol as co-solvent, and the CO_2_ flow rate for the number of total polyphenols and proanthocyanidins in the extracts. The results indicated that the optimization was crucial in order to make a good compromise between the concentration of the valuable compounds of the extracts, and also the length of the extraction process to ensure the economic feasibility for industrial application.

The above summary describes the most common novel extraction techniques that are combined with the enzymatic treatments of the plant materials. However, Ran et al. [206] recently reported the application of the conventional extraction of proanthocyanidins from grape seeds with boosting the extraction with the addition of ionic liquids as adjuvants. The authors reported on the increase of the extraction efficiency when adding ionic liquids. However, they also concluded that further studies are required to benefit fully from the utilization of ionic liquids, including the recovery and reuse of the solvents. On the other hand, the widespread application of ionic liquids in food industrial processes are hindered due to the lack of proper information on their toxicity, and stability and bioactivity limits [207].

When the most promising technological concept is selected for the extraction of bioactive compounds from plant seeds, the extract of the target compounds can undergo downstream processing, such as (pre)concentration by the membrane technology and drying. Shi et al. [208] showed that the concentration of polyphenols with membranes was useful after the extraction with 50% ethanol and 50% water mixtures when a 0.22-µm membrane was applied. Pap et al. [209] also reported on the concentration of anthocyanins and flavonols of blackcurrants in juice by using a reverse osmosis process. An approximate 1.5-fold increase of anthocyanins and flavonols was observed when the process was combined with pectinase enzyme treatment. Conidi et al. [210] successfully implemented a multistep ultrafiltration (150 kDa and 2 kDa membranes) of pomegranate juice to separate anthocyanins from glucose and fructose, and to produce end-products as nutraceuticals and food additives. These findings emphasized the importance of the proper selection of membranes to separate and pre-concentrate the bioactive compounds before the final drying. Due to the heat sensitivity of the bioactives, drying is a challenging step in the downstream processing. For instance, although freeze-drying well preserves the compounds, its widespread industrial application for extract processing will be likely impeded because of the price.

As final remarks, enzyme-assisted extraction, and its combination with novel extraction techniques are gaining importance. There is an increased need to improve the performance of these processes so that they become more resource efficient. In the case of the enzymatic treatment, these should focus on selecting the proper enzyme(s) or blend, and process optimization for operating under the optimal pH, temperature, and enzyme-to-substrate ratio that are defined by their kinetics. The length of the enzymatic treatment is also an important factor to be optimized to reach economic yields. It is also important to consider that chosen enzymes have high stability against the effects that these novel techniques exhibit during the processing. In the case of the novel co-extraction techniques, the operational parameters usually involved are the microwave and ultrasound power, temperatures, pressures, solvent-to-solid ratio, pH, and the extraction time. A target compound is usually determined by the nature of the solvent or solvent mixtures.

As a summary of the extraction technologies, Table 3 [211,212,213,214,215,216,217,218] gathers some studies linking the chemical compounds of fruit seeds and their respective bioactivity with the extracting technology used.

## 4. Final Comments and Upcoming Research Prospects

The potential use of fruit seeds for the recovery of phytochemicals is of pivotal importance within circular economy premises of production and utilization of natural resources. Bioactive-rich extracts may be used for either pharmaceutical or food sectors, and in some cases for both. Future research should focus on the optimization of innovative extraction techniques and on the biological effects of the bioactive-rich extracts using different *in vitro* and *in vivo* protocols. In addition, after checking to ensure that the extracts are safe from the chemical and toxicological standpoints, applications in cosmetics and food models should be conducted to assess the effectiveness of such extracts regarding their antioxidant, antimicrobial, anti-proliferative, antilipidemic, hypoglycemic, and anti-inflammatory effects.

## Figures and Tables

**Table 1:** Chemical compounds and bioactivity of different fruit seeds.

Fruit Seed	Extracting Solvent	Total Phenolic Content	Individual Compounds	Bioactivity	Reference
**Avocado** **(*Persea americana)***	Methanol/chloroform	not determined	Myristic acid Myristoleic Palmitic acid Palmitoleic acid Stearic acid Oleic acid Linoleic acid α-Linolenic acid Eicosadienoic acid Eicosatrienoic acid	DPPH radical scavenging activity ABTS scavenging assay Anti-inflammatory activity Anticancer activity (HepG2 and HCT116)	[51]
	Hydroalcoholic 70% (ethanol)	366.79 ± 5.05 mg GAE/g	Palmitic acid Linoleic acid Oleic acid Stearic acid *p*-Hydroxycoumaroyl quinic acid Caffeoylquinic acid Feruloylquinic acid	Gastroprotective activity	[61]
	Ethanol-water (1:1, *v/v*)	not determined	Vanillic acid 3-Feruloylquinic acid 5-*O*-caffeoylquinic acid Procyanidin dimer B7 Procyanidin pentamer B1 Procyanidin trimer A4 4-*O*-caffeoylquinic acid Procyanidin trimer B2 Procyanidin tetramer B1 Procyanidin dimer B8 Procyanidin pentamer B2 Procyanidin trimer A5 Benzoic acid Procyanidin dimer B9 *p*-Coumaric acid Procyanidin dimer A1 (−)-Epicatechin (Epi)catechin gallate 5-*p*-Coumaroylquinic acid Vanillin 4-*p*-Coumaroylquinic acid Pyrocatechol Procyanidin trimer A1 Procyanidin dimer B3 Syringic acid Procyanidin dimer B4 Procyanidin trimer B1 Tyrosol-glucoside Ferulic acid Procyanidin trimer A2 (Epi)catechin glucopyranoside (isomer 2) 4-Hydroxybenzoic acid Procyanidin tetramer A4 Procyanidin dimer B5 (+)-Catechin Hydroxyferulic acid 3-*p*-Coumaroylquinic acid Gentisic acid Procyanidin trimer A3 Tyrosol-glucosyl-pentoside Procyanidin tetramer A5 Penstemide Procyanidin dimer B Procyanidin dimer B10 Procyanidin trimer A6 Dihydrocaffeic acid Procyanidin dimer A2 Cinchonain (isomer 1) Caffeic acid 4-Feruloylquinic acid Procyanidin dimer A3 Procyanidin trimer A7 Procyanidin dimer A4 Procyanidin dimer B11 Quercetin-diglucoside (isomer 1) Quercetin-diglucoside (isomer 2) Procyanidin dimer A5 Hydroxyabscisic acid glucoside (Epi)gallocatechin Quercetin-3-β-glucoside Ethyl Protocatechuate Cinchonain (isomer 2) Quercetin (±)-Naringenin Sakuranetin	Trolox equivalent antioxidant capacity (TEAC) ABTS scavenging assay DPPH radical scavenging activity	[60]
	Aqueous methanol 80% and aqueous acetone 70%	328.8 ± 13.5 mg GAE/g	Caffeic acid (−)-Epicatechin Vanillin *p*-Coumaric acid Ferulic acid Sinapic acid Procyanidin B4 Quercetin diglucoside Quercetin-3-*O*-arabinosyl-glucoside Quercetin-3-*O*-glucoside Quercetin-3-*O*-rutinoside Quinic acid Procyanidin dimer A Procyanidin trimer B1 Procyanidin dimer B1 Procyanidin trimer B2 Syringic acid Procyanidin dimer B2 (+)-Catechin Procyanidin trimer A Procyanidin dimer B2 Procyanidin trimer B3 5-*O*-Caffeoyl-quinic acid Quercetin Phloridzin Quercetin 3-*O*-rhamnoside Quercetin Apigenin Kaempferol	ABTS scavenging assay DPPH radical scavenging activity	[59]
	Water	45 mg GAE/L	-	ORAC (oxygen radical antioxidant capacity)	[62]
**Grape *(Vitis* sp.)**	Hydroalcoholic 60% (ethylic alcohol)	13643±690 mg GAE/g	Delphinidin-3-glucoside Malvidin-3-glucoside Cyanidin-3-glucoside Malvidin-3,5-diglucoside 2,5 Dihydroxybenzoic acid 2,4 Dihydroxybenzoic acid Syringic acid 5-*O*-Caffeoylquinic acid Gallic acid 2-Hydroxycynnamic acid Ellagic acid Quercetin Quercetin-3-rutinoside (+)-Catechin (-)-Epicatechin *trans*-Resveratrol	Ferric-reducing antioxidant power (FRAP) Folin-Ciocalteu reducing capacity DPPH radical scavenging activity	[64]
	Ethanol/acidic water (pH 3.2) 7:3 *v/v*	not determined	Vanillic acid *p*-Coumaric acid *E*-resveratrol Quercetin Kaempferol Pinoresinol	PTP-1B (Protein Tyrosine Phosphatase 1B enzyme) inhibitory power	[28]
	Ethanol/water (80:20, *v/v*)	161.66 mg GAE/g	Phenolic acid Gallic acid Epicatechin Epigallocatechin gallate Epicatechin gallate Epigallocatechin Procyanidin B1 Procyanidin B4 Procyanidin B2 Flavonol Kaempferol Myricetin Quercetin	Cytotoxicity in murine macrophage RAW 264.7 Anti-inflammatory activity	[65]
	50 mM acetate buffer at 1:10 *w/v*	0.81 g GAE/100g	Gallic acid *p*-Coumaric acid Syringic acid (+)-Catechin Resveratrol Malvidin-3-*O*-glucoside	TEAC	[68]
**Passion fruit** **(*Passiflora* sp)**	Acetonitrile	~40 mg GAE/100g	4-hydroxybenzoic acid Chlorogenic acid Vanillic acid Caffeic acid *p*-Coumaric acid Ferulic acid Rutin Quercetin *trans*-Cinnamic acid	ABTS scavenging assay DPPH radical scavenging activity Gastroprotective activity	[70]
	Water	0.14 mg GAE/mL	Vanillic acid Syringic acid Gallic acid Rutin Quercetin	ORAC and DPPH assays Folin-Ciocalteu assay	[66]
	Hexane Ethyl acetate Ethanol Ethanol/water	Maceration (EtOH–H_2_O 142.4 ± 0.4 mg GAE/g, EtOH 75 ± 2 mg GAE/g, EtOAc 24.6 ± 0.8 mg, GAE/g, Hx 30 ± 1 mg GAE/g) Ultrasound-assisted (EtOH–H2O 61.3 ± 0.4 mg GAE/g, EtOH 21.4 ± 0.4 mg GAE/g, EtOAc 19 ± 1 mg GAE/g, Hx 22.3 ± 0.1 mg GAE/g) Supercritical fluid extraction (40 °C 150 bar 33.0 ± 0.8 mg GAE/g, 250 bar 30 ± 1 mg GAE/g, 300 bar 18 ± 2 mg GAE/g, 50 °C 150 bar 26.4 ± 0.8 mg GAE/g, 250 bar 24.3 ± 0.4 mg GAE/g, 300 bar 19.3 ± 0.8 mg GAE/g)	not determined	ABTS scavenging assay DPPH radical scavenging activity Antimicrobial activity	[69]
	Petroleum ether Chloroform Acetone Methanol	115.13 ± 8.42 mg GAE/g, 113.45 ± 6.19 mg GAE/g, 417.65 ± 7.33 mg GAE/g, 227.17 ± 10.97 mg GAE/g	Quercetin Gallic acid Apigenin Catechin	DPPH FRAP Metal chelating activity Superoxide radical scavenging activity Analgesic activity Anti-inflammatory activity Antipyretic effect	[67]
**Pomegranate (*Punica granatum*)**	Ethanolic extracts - soluble and insoluble-bound phenolic compounds	3.1 ± 0.3 (mg GAE/g)	not determined	ORAC and TEAC assays	[41]
	Ethanolic extracts	73 ± 13.35 (mg GAE/g)	not determined	Folin-Ciocalteu reducing capacity and β-Carotene oxidation method	[42]
	Methanolic extracts (oil)	88.45 ± 3.89 mg GAE/kg oil	not determined	DPPH	[43]
	Isolation of free, esterified and insoluble-bound phenolic compounds	Free 1.38 ± 0.01 Esterified 1.39 ± 0.01 and Insoluble-bound 0.62 ± 0.01 (mg GAE/g)	Gallic acid (major phenolic acid present)	TEAC, electron paramagnetic resonance (EPR) spectrometry, Metal chelating ability	[44]
	Ethanolic extracts (oil)	not determined	Palmitic (16:0) stearic (18:0) oleic (18:1 ω-9) linoleic (18:2 ω-6) acids	β-carotene bleaching assays, DPPH, ORAC, and ABTS assays	[45]
**Camu-camu (*Myrciaria dubia*)**	aqueous acetone (50:50 *v/v*)	369.4±9.6 (mg GAE/g)	not determined	ABTS scavenging assay DPPH radical scavenging activity	[46]
	Ethanolic extracts	3738.0 ± 20.8 mg GAE/100 g	Ellagic acid Syringic acid Quercetin Myricetin Catechin	DPPH, Folin–Ciocalteau reducing capacity	[47]
	Aqueous extracts	not detected	not determined	FRAP and DPPH assays	[48]
	Ethanolic extracts	128 mg GAE/100 g	Rosmarinic acid 2,4-dihydroxybenzoic acid Ellagic acid Cyanidin-3-glucoside Methylvescalagin *trans*-Resveratrol Quercetin	DPPH, FRAP and Folin-Ciocalteu reducing capacity	[49]
	EtOH: ethyl alcohol, H_2_O: water, (CH_3_)_2_CO: propanone)	2400 (EtOH), 4000 (H_2_O) and 1300 ((CH_3_)_2_CO) mg GAE/100 g	Gallic and chlorogenic acid (H_2_O) Ellagic acid (EtOH) Ferulic acid ((CH_3_)_2_CO)	DPPH, FRAP and Folin-Ciocalteu reducing capacity assays	[15]
**Jabuticaba (*Plinia jaboticaba* and *Plinia cauliflora*)**	Ethanolic extracts	116.17 ± 7.10 mg of GAE/ g	not determined	not determined	[53]
	Soluble phenolics, Alkaline hydrolysis and Acid hydrolysis	not determined	Cyanidin-3-*O*-glucoside Gallic acid Delphinidin-3-*O*-glucoside Rutin	Folin–Ciocalteu, FRAP, TEAC and ORAC assays	[54]
	water:propanone (60:40 *v/v*)	8.65 g GAE/100 g	Ellagic acid and ellagitannins	DPPH scavenging activity and total reducing capacity	[55]
	Methanolic extracts	not determined	Ellagic acid derivatives	Folin–Ciocalteu reducing capacity, DPPH and FRAP assays	[56]
**Apple *(Malus domestica)***	Methanol	5.74–17.44 GAE/g	Protocatechuic acid (+)-Catechin Proanthocyanin B2 Chlorogenic acid Caffeic acid (-)-Epicatechin Quercetin Hyperin Phloridzin	DPPH, FRAP and ABTS assays	[50]
	Aqueous acetone (30:70; *v/v*) and ethyl acetate extraction after hexane extraction	not determined	3-*p*-Coumaroylquinic acid 5-Caffeoylquinic acid 4-Caffeoylquinic acid Caffeic acid 3-Caffeoylquinic acid 5-*p*-Coumaroylquinic acid 4-*p*-Coumaroylquinic acid Proanthocyanin B2 Epicatechin 3-hydroxyphloridzin Phloretin-xyloglucoside Phloridzin Phloretin Procyanidins	not determined	[52]
	Ethanol-water (30:70)	not determined	Phloridzin	not determined	[58]
	Methanol	not determined	Quercetin Quercetin-3-*O*-galactoside Quercetin-3-*O*-glucoside Quercetin-3-*O*-rhamnoside Phloridzin Phloretin (+)-Catechin (-)-Epicatechin Cyanidin-3-*O*-galactoside Luteolin-7-*O*-glucoside Caffeic acid Chlorogenic acid Ferulic acid Isoferulic acid	not determined	[57]
	Methanol	not determined	Epicatechin Procyanidin B2 Catechin Chlorogenic acid *p*-Coumaroylquinic acid Quercetin-3-*O*-galactoside Quercetin-3-*O*-rhamnoside Quercetin-3-*O*-glucoside Phloridzin Phloretin-xyloglucoside Phloretin	not determined	[71]
	Hexane and 70% aqueous acetone after hexane extraction	not determined	Linoleic acid Palmitic acid Linolenic acid Stearic acid Oleic acid Chlorogenic acid *p*-Coumaroylquinic acid Quercetin-3-galactoside Quercetin-3-rutinoside Quercetin-3-glucoside 3-hydroxyphloridzin Quercetin-3-xyloside Phloretin-2′-xyloglucoside Quercetin-3-arabinoside Quercetin-3-rhamnoside Phloridzin	not determined	[72]
**Plum (*Prunus* sp.)**	Aqueous methyl alcohol (50%) and aqueous acetone (70%)	Total extractable polyphenols 86 mg gallic acid/100 g		ABTS assay	[63]
		Total carotenoids 3.9 μg/g			
	*n*-hexane and ultrasonication		α-Tocopherol		[73]
			β-Tocopherol		
			γ-Tocopherol		
			δ-Tocopherol		
			α-Tocotrienol		
			γ-Tocotrienol		
			Cholesterol		
			Campesterol		
			Δ5-Stigmasterol		
			β-Sitosterol		
			Sitostanol		
			Δ5-Avenasterol		
			Δ7-Stigmasterol		
			Cycloartenol		

**Table 2:** Previous research on phenolic compounds and bioactivity of berry seeds.

Fruit Seeds	Extraction Solvents	Total Phenolics ^a^	Individual Phenolics	Bioactivity ^b^	Reference
**Bilberry**					
*Vaccinium myrtillus*	water	2.3 mg GAE/g dry extract	not determined	**Anti-oxidative activity** TEAC (0.1 and 82 μmol TE/g dry extract in *n*-hexane and distilled water, respectively)	[159]
seed oil, *Vaccinium myrtillus*		not determined	not determined	**Anti-oxidative activity** OOR• scavenging (519 μmol α-tocopherol equivalent/100 g oil), Inhibition of microsomal lipid peroxidation (1188 μmol trolox equivalent/100 g oil)	[163]
seed oil, *Vaccinium myrtillus*		not determined	not determined	**Antioxidant activity** DPPH (EC_50_ = 5.5–9.5 mg oil/mL)	[164]
**Blackberry**					
*Rubus* spp.	methanol:acetone:water (7:7:6, *v/v/v*), acetone:water (8:2, *v/v*), methanol:water (7:3, *v/v*), water, diethyl ether:ethyl acetate (1:1, *v/v*)	13 mg GAE/g dry extract	protocatechuic acid, *p*-coumaric acid, gallic acid, gallic hexoside, caffeic acid, syringic acid, catechin, epicatechin, epigallocatechin, epicatechin gallate, B-type procyanidin dimmers, quercetin, quercetin 3-*O*-glucoronide, quercetin pentose, myricetin, peonidin 3-*O*-glucoside	**Antioxidant activity** OH• scavenging (125 μmol TE/g dry extract), ORAC (60 μmol TE/g dry extract), Reducing power (88 μmol TE/g dry extract), Inhibition against human LDL-cholesterol oxidation (21–60% from 0 to 22 h), Iron(II) chelating (199 μmol TE/g dry extract), Inhibition of OH• and OOR• induced supercoiled DNA strand scission (85–95% for OH•, and 90–95% for OOR•)	[19]
*Rubus ulmifolius*	methanol	171 mg GAE/g dry seeds	cyanidin 3-*O*-glucoside, ellagic acid, galloyl-HHDP-glucose, galloyl-bis-HHDP-glucose	**Antioxidant activity** DPPH (93, 96, and 98% radicals scavenged at 42, 83, 167 μg/mL of extracts, respectively; EC_50_ = 1 μg/mL) **Anti-inflammatory activity** Strong inhibitory effects on the production of LPS-induced inflammatory mediators (NO, CCL-20)	[165]
*Rubus fruticosus* 3 cultivars	methanol:water (1:1, *v/v*)	not determined	Ellagitannins (41 compounds), ellagic acid derivatives (10), gallic acid derivatives (4), protocatechuic acid, chlorogenic acid, salicylic acid	**Protective effect on chromosome aberrations in peripheral human lymphocytes**	[166]
seed oil, *Rubus fruticosus*	methanol:water (1:1, *v/v*)	5.6–9.1 mg CAE/g oil	*p*-coumaric acid, vanillic acid, vanillin	**Antioxidant activity** FRAP (0.3 FeSO_4_ equivalent (μmol/L)/g oil)	[167]
**Blueberry**					
*Vaccinium* spp.	methanol:acetone:water (7:7:6, *v/v/v*), acetone:water (8:2, *v/v*), methanol:water (7:3, *v/v*), water, diethyl ether:ethyl acetate (1:1, *v/v*)	2 mg GAE/g dry extract	protocatechuic acid, *p*-coumaric acid, gallic acid, gallic hexoside, caffeic acid, syringic acid, catechin, epicatechin, epigallocatechin, epicatechin gallate, B-type procyanidin dimmers, quercetin, quercetin pentose, myricetin, kaempferol hexoside, delphinidin 3-*O*-hexoside, cyanidin 3-*O*-(6-*O*-acetyl)galactoside, peonidin 3-*O*-glucoside, peonidin 3-*O*-arabinoside, petunidin 3-*O*-galactoside, petunidin 3-*O*-arabinoside	**Antioxidant activity** OH• scavenging (37 μmol TE/g dry extract), ORAC (5 μmol TE/g dry extract), Reducing power (60 μmol TE/g dry extract), Inhibition against human LDL-cholesterol oxidation (6–49% from 0 to 22 h), Iron(II) chelating (12 μmol TE/g dry extract), Inhibition of OH• and OOR• induced supercoiled DNA strand scission (50–70% for OH•, and 55–75% for OOR•)	[19]
seed oil, *Vaccinium corymbosum*	methanol:water (1:1, *v/v*)	8.8–9.5 mg CAE/g oil	homovanillic acid, vanillin	**Antioxidant activity** FRAP (0.26–0.41 FeSO_4_ equivalent (μmol/L)/g oil)	[167]
seed flour, *Vaccinium corymbosum*	acetone:water (1:1, *v/v*)	16 mg GAE/g seed flour	not determined	**Antioxidant activity** ORAC (153 μmol TE/g seed flour), DPPH (ED_50_ = 670 μg flour equivalents/mL), Iron(II) chelating (1.9 mg EDTA equivalents/g seed flour)	[168]
**Cloudberry**					
*Rubus chamaemorus*, after fermentation	acetone:water (7:3, *v/v*)	457 mg/g dry extracts (measured by HPLC)	ellagic acid, ellagic acid glycosides, sanguiin H10, casuarictin/potentillin, lambertianin C, sanguiin H6, sanguiin H2, ferulic acid, quercetin 3-*O*-[6″-(3-hydroxy- 3-methylglutaroyl)-β-glucoside], quercetin-3-*O*-glucuronide	**Anti-bacterial activity** *Staphylococcus aureus* (very strong inhibition), *Escherichia coli* (strong inhibition), *Pseudomonas aeruginosa* (clear inhibition), *Candida albicans* (no inhibitory effects), *Saccharomyces cerevisiae* (no inhibitory effects) **Anti-inflammatory activity** significantly reduced NO and IL-6 production and iNOS expression in activated macrophages	[171]
seed oil, *Rubus chamaemorus*		not determined	not determined	**Antioxidant activity** OOR• scavenging (1157 μmol α-tocopherol equivalent/100 g oil), Inhibition of microsomal lipid peroxidation (650 μmol trolox equivalent/100 g oil)	[163]
**Cranberry**					
*Vaccinium oxycoccus*	water	not determined	not determined	**Antioxidant activity** TEAC (0.1 and 50 μmol TE/g dry extract in *n*-hexane and distilled water, respectively)	[159]
seed oil, *Vaccinium macrocarpon*	methanol:water (1:1, *v/v*)	11.0–11.3 mg CAE/g oil	4-(2-hydroxyethyl)phenol, p-coumaric acid, homovanillic acid, vanillic acid, protocatechuic acid	**Antioxidant activity** FRAP (0.27–0.29 FeSO_4_ equivalent (μmol/L)/g oil)	[167]
seed flour, *Vaccinium macrocarpon*	acetone:water (1:1, *v/v*)	15 mg GAE/g seed flour	not determined	**Antioxidant activity** ORAC (111 μmol TE/g seed flour), DPPH (ED_50_ = 1260 μg flour equivalents/mL), Iron(II) chelating (2.1 mg EDTA equivalents/g seed flour) **Anti-proliferative activity** significant inhibited HT-29 cell proliferation	[168]
seed oil, *Vaccinium oxycoccos*		not determined	not determined	**Antioxidant activity** OOR• scavenging (1543 μmol α-tocopherol equivalent/100 g oil), Inhibition of microsomal lipid peroxidation (399 μmol trolox equivalent/100 g oil)	[163]
seed oil, *Vaccinium macrocarpon*		not determined	not determined	**Antioxidant activity** OOR• scavenging (975 μmol α-tocopherol equivalent/100 g oil), Inhibition of microsomal lipid peroxidation (338 μmol trolox equivalent/100 g oil)	[163]
**Currant**					
black, *Ribes nigrum* 5 cultivars	acetone:water (1:1, *v/v*)	1.6–2.3 mg GAE/g dry seed residues	delphinidin 3-*O*-glucoside, delphinidin 3-*O*-rutinoside, cyanidin 3-*O*-glucoside, cyanidin 3-*O*-rutinoside, myricetin 3-*O*-glucoside, myricetin 3-*O*-rutinoside, quercetin 3-*O*-glucoside, quercetin 3-*O*-rutinoside, kaempferol 3-*O*-glucoside, kaempferol 3-*O*-rutinoside, *p*-coumaric acid, *p*-coumaroyl glycoside	**Antioxidant activity** ABTS (14–17 μmol TE/g dry seed residues), DPPH (11–13 μmol TE/g dry seed residues)	[170]
black, *Ribes nigrum*	water	0.9–1.8 mg GAE/g dry extract	not determined	**Antioxidant activity** TEAC (0.6–0.7 and 26–60 μmol TE/g dry extract in *n*-hexane and distilled water, respectively)	[159]
black, seed oil, *Ribes nigrum*		not determined	not determined	**Antioxidant activity** OOR• scavenging (1068 μmol α-tocopherol equivalent/100 g oil), Inhibition of microsomal lipid peroxidation (478 μmol trolox equivalent/100 g oil)	[163]
**Elderberry**					
*Sambucus nigra*	methanol	54 mg GAE/g dry seeds	cyaniding 3-*O*-sambubioside -5-*O*-glucoside, pelargonidin 3-*O*-rutinoside, quercetin 3-*O*-rutinoside, quercetin 3-*O*-glucoside	**Antioxidant activity** DPPH (42, 55, 82% radicals scavenged at 42, 83, 167 μg/mL of extracts, respectively; EC_50_ = 82 μg/mL) **Anti-inflammatory activity** No inhibitory effects on the production of LPS-induced inflammatory mediators (NO, CCL-20)	[165]
*Sambucus nigra*	water	1.5 mg GAE/g dry extract	not determined	**Antioxidant activity** TEAC (0.8 and 30 μmol TE/g dry extract in *n*-hexane and distilled water, respectively)	[159]
**Lingonberry**					
seed oil, *Vaccinium vitis-idaea*		not determined	not determined	**Antioxidant activity** OOR• scavenging (299 μmol α-tocopherol equivalent/100 g oil), Inhibition of microsomal lipid peroxidation (329 μmol trolox equivalent/100 g oil)	[163]
**Raspberry**					
black, *Rubus* spp.	methanol:acetone:water (7:7:6, *v/v/v*), acetone:water (8:2, *v/v*), methanol:water (7:3, *v/v*), water, diethyl ether:ethyl acetate (1:1, *v/v*)	7 mg GAE/g dry extract	protocatechuic acid, *p*-coumaric acid, gallic acid, gallic hexoside, caffeic acid, syringic acid, catechin, epicatechin, epigallocatechin, epicatechin gallate, B-type procyanidin dimmers, quercetin, quercetin 3-*O*-glucoronide, quercetin pentose, myricetin, peonidin 3-*O*-glucoside	**Antioxidant activity** OH• scavenging (67 μmol TE/g dry extract), ORAC (47 μmol TE/g dry extract), Reducing power (60 μmol TE/g dry extract), Inhibition against human LDL-cholesterol oxidation (18–57% from 0 to 22 h), Iron(II) chelating (69 μmol TE/g dry extract), Inhibition of OH• and OOR• induced supercoiled DNA strand scission (60-80% for OH•, and 80-85% for OOR•)	[19]
seed oil, *Rubus* spp.	methanol	2.7 mg CAE/100 g oil	not determined	**Antioxidant activity** Inhibitory effects on the activities of superoxide dismutase and glutathione peroxidase	[160]
red, seed oil, *Rubus idaeus*	methanol:water (1:1, *v/v*)	8.4 mg CAE/g oil	4-(2-hydroxyethyl)phenol	**Antioxidant activity** FRAP (0.34 FeSO_4_ equivalent (μmol/L)/g oil)	[167]
black, seed flour, *Rubus occidentalis*	acetone:water (1:1, *v/v*)	41 mg GAE/g seed flour	not determined	**Antioxidant activity** ORAC (296 μmol TE/g seed flour), DPPH (ED_50_ = 200 μg flour equivalents/mL), Iron(II) chelating (3.6 mg EDTA equivalents/g seed flour) **Anti-proliferative activity** significant inhibited HT-29 cell proliferation	[168]
red, seed flour, *Rubus ideaus*	acetone:water (1:1, *v/v*)	25 mg GAE/g seed flour	not determined	**Antioxidant activity** ORAC (276 μmol TE/g seed flour), DPPH (ED_50_ = 510 μg flour equivalents/mL), Iron(II) chelating (3.9 mg EDTA equivalents/g seed flour)	[168]
seed oil, *Rubus idaeus*		not determined	not determined	**Antioxidant activity** OOR• scavenging (2315 μmol α-tocopherol equivalent/100 g oil), Inhibition of microsomal lipid peroxidation (424 μmol trolox equivalent/100 g oil)	[163]
**Sea buckthorn**					
*Hippophaë rhamnoides*	ethanol:water (1:9, *v/v*)	120 mg GAE/g dry extract	tannins, gallic acid, quercetin 3-*O*-galactoside, kaempferol, isorhamnetin, isorhamnetin 3-*O*-glucoside, isorhamnetin 3-*O*-rutinoside	**Antioxidant activity** DPPH (529 mg TE/g dry extract), FRAP (454 mg TE/g dry extract) **Anti-bacterial activity** *Enterecoccus durans* (68% inhibition percentage), *Candida albicans* (68%), *Bacillus cereus* (64%), *Staphylococcus aureus* (41%), *Escherichia coli* (38%), *Pseudomonas aeruginosa* (28%)	[161]
*Hippophaë rhamnoides*	ethanol	9.4–23.5 mg GAE/g dry seeds	quercetin-3-*O*-galactoside, quercetin, myricetin, isorhamnetin	**Antioxidant activity** ABTS (44–182 μmol TE/g dry seeds), DPPH (128–283 μmol TE/g dry seeds)	[162]
seed oil, *Hippophaë rhamnoides*		not determined	not determined	**Antioxidant activity** OOR• scavenging (1323 μmol α-tocopherol equivalent/100 g oil), Inhibition of microsomal lipid peroxidation (245 μmol trolox equivalent/100 g oil), Inhibition of LDL oxidation (IC_50_ = 2.0 μL oil/mg LDL), O_2_^•-^ scavenging (IC_50_ = 2 g oil/L), Inhibition against DNA oxidation (protective effects on purified DNA and rat liver homogenate from UV-induced DNA oxidation *in vitro*)	[163]
**Strawberry**					
seed oil, *Fragaria* × *ananassa*	methanol	1.8 mg CAE/100 g oils	not determined	**Antioxidant activity** Inhibitory effects on the activities of superoxide dismutase and glutathione peroxidase	[160]
*Fragaria* × *ananassa*	water	1.7 mg GAE/g dry extract	not determined	not determined	[159]
seed oil, *Fragaria* × *ananassa*	methanol:water (1:1, *v/v*)	9.3–10.4 mg CAE/g oil	*p*-coumaric acid, ferulic acid, 4-(2-hydroxyethyl)phenol, homovanillic acid, vanillic acid, vanillin.	**Antioxidant activity** FRAP (0.24–0.30 FeSO_4_ equivalent (μmol/L)/g oil)	[167]
seed oil, *Fragaria* × *ananassa*		not determined	not determined	**Antioxidant activity** OOR• scavenging (296 μmol α-tocopherol equivalent/100 g oil), Inhibition of microsomal lipid peroxidation (1015 μmol trolox equivalent/100 g oil)	[163]

Note: ^a^ GAE means gallic acid equivalents, CAE means caffeic acid equivalents; ^b^ TE means trolox equivalents.

**Table 3:** Examples of extractions of bioactive compound from apple pomace.

Extraction	Agro-Industrial Waste	Total Bioactive Compounds	Individual Bioactive Compounds	Antioxidant Activity (AA)	Conclusion Remarks	Reference
Conventional extraction: 30 °C for 30 min in a shaker 70% with methanol containing 2% formic acid	Apple pomace (peel and seed)	Total phenols (mg GAE/L) Peel: 2228.49 Pomace: 208.75 Total flavanols (mg CTE/L) Peel: 170.33 Pomace: 40.91	Phenolic compounds (mg/L) Peel: Gallic acid: 40.42 Vanillic acid: 58.70 Caffeic acid: 0.21 Chlorogenic acid: 7.05 Catechin: 37.67 Epicatechin gallate: 12.85 Phlorizin: 60.28 Pomace: Gallic acid: 29.57 Vanillic acid: 56.02 Chlorogenic acid: 5.29 Catechin: 35.40 Epicatechin gallate: 21.68 Phlorizin: 12.81	DPPH (µmol TE/L) Peels: 4203.59 Pomace: 3830.72	Peel pomace contained significantly higher amounts of bioactive compounds in comparison to pulp pomace. Due to high levels of antioxidant and bioactive compounds, apple pomaces could be a better material to make apple cider vinegar.	[211]
Conventional extraction: Magnetic stirring with methanol during 30 min. After centrifugation and filtration, methanol was evaporated through Soxhlet extraction (60 ± 0.5 °C). Crude extract was further dissolved and extracted with ethanol, chloroform, ethyl acetate and n-hexane to obtain their fractions.	Apple pomace freeze-dried and milled	Total phenols (mg GAE/g DW) Methanol: 3.48 Ethanol: 3.04 Ethyl acetate: 2.85 Chloroform: 1.64 n-hexane: 1.50	The average amounts of 6 triterpenoic acids detected (mg/100 g DW): maslinic acid: 0.96 oleanolic acid: 3.18 ursolic acid: 6.14 betulinic acid: 1.78 erythrodiol: 0.78 uvaol: 0.84	DPPH (%) Methanol: 72.6 Ethanol: 67.5 Ethyl acetate: 64.2 Chloroform: 59.6 n-hexane: 56.2 FRAP (%) Methanol: 65.8 Ethanol: 60.3 Ethyl acetate: 58.3 Chloroform: 55.2 n-hexane: 50.1 ABTS (%) Methanol: 84.3 Ethanol: 79.2 Ethyl acetate: 72.9 Chloroform: 65.3 n-hexane: 60.8	The antioxidant activity results showed that the methanol and ethanol fractions were more effective radical scavengers than chloroform, ethyl acetate and n-hexane. Both alcoholic fractions showed potential toward tyrosinase, xanthine oxidase and urease inhibition. Among the TAAs, ursolic acid, betulinic acid and maslinic acid showed effective radical scavenging activity. All TAAs showed lower inhibitory activity towards free radicals *in vitro* than the apple pomace extracts. Apple pomace methanol extract and ursolic acid revealed prominent anticancer activity on Hela, Skov-3, Caski, and NCL cancer cell lines, respectively.	[212]
Ultrasound Assisted Extraction (UAE): Defatted seed flours were mixed with 80% methanol and held in an ultrasonic bath for 30 min at room temperature.	Apple seeds (defatted seed flours)	Total phenols (mg GAE/kg DS) Fuji Zhen Aztec 2861 Granny Smith 3581 Jeromine 4096 Pink Lady 3616 Super Chief 5141	Phenolic compounds (mg/kg DM): Fuji Zhen Aztec Phloridzin: 1748.7 Ellagic acid: 189.5 (-)-Epicatechin: 76.3 Caffeic acid: 114.2 (+)-Catechin: 90.1 Ferulic acid: 57.8 Protocatechuic acid: 46.8 Gallic acid: 4.2 Granny Smith Phloridzin: 2106.6 Ellagic acid: 275.3 (-)-Epicatechin: 77.2 Caffeic acid: 9.1 (+)-Catechin: 5.0 Ferulic acid: 142.2 Protocatechuic acid: 48.7 Gallic acid: 7.5 Jeromine Phloridzin: 2623.5 Ellagic acid: 286.7 (-)-Epicatechin: 164.6 Caffeic acid: 11.8 (+)-Catechin: N.D. Ferulic acid: 21.2 Protocatechuic acid: 153.8 Gallic acid: 5.6 Pink Lady Phloridzin: 1888.4 Ellagic acid: 216.5 (-)-Epicatechin: 73.9 Caffeic acid: 10.4 (+)-Catechin: 21.0 Ferulic acid: 8.2 Protocatechuic acid: 39.6 Gallic acid: 4.2 Super Chief Phloridzin: 3462.2 Ellagic acid: 230.7 (-)-Epicatechin: 69.0 Caffeic acid: 14.2 (+)-Catechin: 191.0 Ferulic acid: 36.4 Protocatechuic acid: 161.3 Gallic acid: 7.9	ABTS (mg GAE/kg DS) Fuji Zhen Aztec 363 Granny Smith 292 Jeromine 368 Pink Lady 392 Super Chief 343 mg DPPH µmol TE/g DS) Fuji Zhen Aztec 24.28 Granny Smith 33.12 Jeromine 32.04 Pink Lady 21.45 Super Chief 43.56	Phloridzin represented 52–67% and 75–83% of the total phenolics measured by the Folin-Ciocalteu assay and HPLC method, respectively. Chewing gum could be a suitable delivering material for phloridzin uptake originated from apple seeds. 5 min was enough for the dissolution of almost all added phloridzin (88.43–96%).	[213]
Hot water extraction (HWE): Boiling water with 1% acetic acid at S/L=1/60 g/mL during 10 min.	Apple pomace obtained after processing of a	Total phenols (µg GAE of extract/mL) HWE: 10.7 pHWE: 149	pHWE was mainly composed of flavonols: quercetin-3-*O*-galactoside (27%), quercetin-3-*O*- rhamnoside (23%), quercetin- 3-*O*-arabinofuranoside (13%), and	DPPH (EC_50_, µg of extract/mL) HWE: 1339 pHWE: 82.4 ABTS (EC_50_, µg of extract/mL)	HWE represented 29% of dry apple pomace and presented 11 g/kg of polyphenols. 3.26 g GAE of polyphenols per kg of apple pomace were extracted.	
Fractionation processes of HWE extracts: ultrafiltration to obtain LMWM (low molecular weight material) and HMWM (high molecular weight material); SPE to obtain polyphenol-isolated fraction (pHWE) and non-retained material (NrFr).	mixture of apples (Royal Gala variety), employing milling, enzymatic digestion and pressing processes, afterwards it was freeze-dried.	Ultrafiltration: 11% of the polyphenols from the HWE remained in the HMWM. This fraction accounted for 6.9% of the apple pomace. SPE: The hydrophobic fraction pHWE corresponded to 1.6% of the dry apple pomace and accounted for 63% of those in the HWE.	the dihydrochalcone phloretin-2-*O*-glucoside (14%).	HWE: 532 pHWE: 35.2	When apple pomace HWE were added to yogurt formulations, a final product with improved fiber content and antioxidant properties was achieved in comparison to control sample (plain yogurt).	[214]
Ultrasound Assisted Extraction (UAE): Extraction with aqueous acetone (70/30 vol) for 5 min in an ultrasonic bath at 20°C.	Apple seeds oil and defatted seed flour from the cider- making industry	In defatted seeds flour: Total phenols 2.7-6.7 mg GAE/g defatted matter Condensed tannins 2.4-4.0 mg cyanidin/g defatted matter Hydrolysable tannins 34.5-47.2 mg GAE/g defatted matter		In apple seeds oil: DPPH IC_50_ = 4.8-5.8 mg of oil/mL	Apple seeds oil exhibited an significant antioxidant activity due to high levels of tocopherols (1280 ± 104.8 mg/kg oil) with β-tocopherol (794.5 ± 62.2 mg/kg oil) being most abundant, followed by α-tocopherol (439.2 ± 34.5 mg/kg oil). Defatted seeds flour contained considerable amounts of phenolic compounds.	[215]
Microwave-assisted extraction (MAE): MAE parameters: solvent type: 70% acetone (Ac), 60% ethanol (Et); microwave power: 100–900W; solvent volume to sample ratio: 4–12 mL/g dry pomace; extraction time: 30–180 s.	Freeze-dried apple pomace of Red Delicious (RD) and Jonathan (J) apple varieties	Total phenols (mg GAE/g DW) under the optimum MAE parameters: RD-Ac: 6.66 RD-Et: 15.81 J-Ac: 5.79 J-Et: 7.65	Phenolic compounds (µg/g dry pomace) Phloridzin RD-Ac: 82.59 RD-Et: 25.25 J-Ac: 102.9 J-Et: 79.80 Gallic acid: RD-Ac: nd RD-Et: 189.09 J-Ac: nd J-Et: 23.08 Caffeic acid: RD-Ac: 10.00 RD-Et: 27.63 J-Ac: 9.77 J-Et: 34.67 Chlorogenic acid: RD-Ac: nd RD-Et: nd J-Ac: nd J-Et: 22.56 Quercetin: RD-Ac: 46.33 RD-Et: 59.20 J-Ac: 115.15 J-Et: 211.34	DPPH (%) 60% ethanol: 77.1 70% acetone: 93.7	The optimum conditions for MAE: 735 W power and 149 s extraction time with 10.3 mL of ethanol per gram dry sample. Higher total phenols and DPPH radical scavenging activity was found in Red Delicious apple pomace extracts. Extraction solvent was not found as significant extraction parameter.	[216]
Conventional extraction (CE): agitation (1000 rpm) of samples (2.5 g) in water (50 mL) at room temperature. Ultrasound-assisted extraction (UAE): 10 g of sample in 200 mL of water ultrasonic (US) probe diameter: 1.2 cm, height: 13.7 cm at a depth of ca. 20 mm. US power: 7.8 or 49.5 W The aliquots were sampled every 2, 5, 10, 20 and 30 min and stored until further analysis. Microwave-assisted extraction (MAE): 10 g of sample in 200 mL of water. Power: 400, 600, 1000 W (which corresponds to the actual power of 302, 384, and 435 W) for 2 or 4 irradiation cycles, each consisted of 1 or 1.5 min. High speed homogenization (HH)+MAE: Samples were pre-treated with a high-speed homogenizer prior to MAE. Homogenization at 22,000 rpm for 1 min + MAE: 1000 W for 4 cycles of 1.5 min each. Ultrasound-assisted enzymatic extraction (UAEE): the same as UAE + the pectinase solution	Apple pomace consisted of stalk, skin and the fruit kernel were mixed thoroughly and ground into powder.	Total phenols (mg GAE/g raw sample) CE: 2.62-2.68 UAE: 0.7-2.11 MAE: 2.23-2.81 HH: 2.32-2.44 HH+MAE: 2.65-2.78 UAEE: 2.50-4.62	LC-MS analysis of fractions obtained from UAEE revealed the presence of 26 phenolic compounds.	The DPPH scavenging ability of the extracts ranged from 27.1 to 54.6 ± 6.9 mg trolox/L, depending on the extraction parameters.	In most cases, extraction time and the particle size of the sample had significant effect on the extraction yield and longer extraction times lead to increased TPC in the extract. UAEE found to be superior techniques to extract TPC from apple pomace due to the synergistic effects of US and enzymes. The antioxidant activity decreased with the extraction time and enzyme dosage.	[217]
Mild intensity pulsed electric field (PEF) PEF treatment: 1, 2 and 3 kV/cm 500, 875 and 1250 μs The specific energy intake: 0.44-17.0 kJ/kg Extraction: Fluor to water ratio (FWR): 5, 8.75, 12.5% (*w/v*) Centrifugation with methanol. Free phenolic acid extraction: shaking with 80% aqueous methanol, centrifugation, evaporation and	Fermented apple pomace powder (12.5% *w/v*).	Total phenols 402.7 mg GAE/100g DW (at 12.5% FWR)	Phenolic compounds (μg/g DW) Control samples: Protocatechuic acid: 392.6 Chlorogenic acid: 72.5 Caffeic acid: 130.8 *p*-Coumaric acid: 24.5 Ferulic acid: 64.4 Salicylic acid: 131.9 PEF treated samples: Protocatechuic acid: 98.9 Caffeic acid: 141.1 *p*-Coumaric acid: 34.3 Ferulic acid: 70.1 Salicylic acid: 170.2	DPPH 799.3 μmol TE/100g DW (at 12.5% FWR)	Total phenols and AA were significantly (*p* < 0.05) influenced by the FWR and its interaction with the electric filed intensity and time. With the increase in electric filed intensity from 1 kV/cm to 3 kV/cm, a significant (*p* < 0.05) increase of 4.8% TPC and 4.4% AA was observed. A significant (*p* < 0.05) decrease of 3.8% and 2.6% was observed in total phenols and AA, respectively with the increase in treatment time from 500 μs to 875 μs followed by no significant (p > 0.05) change in both total phenols and AA. The optimum condition of PEF treatment was found as 12.5% (*w/v*) FWR, 2 kV/cm electric field intensity and 500 μs treatment time with a specific energy input of 3.0 kJ/kg. Obtained results indicated that the PEF treatment could be useful tool for the processing of food with enhanced levels of phenolic antioxidants.	[218]
